# The dynamics of memory retrieval for internal mentation

**DOI:** 10.1038/s41598-019-50439-y

**Published:** 2019-09-26

**Authors:** David Stawarczyk, Arnaud D’Argembeau

**Affiliations:** 1 Department of Psychology, Psychology and Neuroscience of Cognition Research Unit, University of Liège, 4000 Liège, Belgium; 2 GIGA-CRC In Vivo Imaging, University of Liège, 4000 Liège, Belgium

**Keywords:** Long-term memory, Human behaviour

## Abstract

Daily life situations often require people to remember internal mentation, such as their future plans or interpretations of events. Little is known, however, about the principles that govern memory for thoughts experienced during real-world events. In particular, it remains unknown whether factors that structure the retrieval of external stimuli also apply to thought recall, and whether some thought features affect their accessibility in memory. To examine these questions, we asked participants to undertake a walk on a university campus while wearing a lifelogging camera. They then received unexpected recall tasks about the thoughts they experienced during the walk, rated the phenomenological features of retrieved thoughts, and indicated the moment when they were experienced. Results showed that thought retrieval demonstrates primacy, recency, and temporal contiguity effects, and is also influenced by event boundaries. In addition, thoughts that involved planning and that were recurrent during the walk were more accessible in memory. Together, these results shed new light on the principles that govern memory for internal mentation and suggest that at least partially similar processes structure the retrieval of thoughts and stimuli from the external environment.

## Introduction

Imagine that you are driving back home after work when you suddenly think of a novel experiment to include in the grant proposal you are currently writing. You have no means to take notes and will therefore have to rely on your memory to write down these ideas at a more appropriate time, for instance when arriving at home. Now imagine that later that night you finish reading a mystery novel that has kept you occupied in the last few days. The following morning this book comes up in a discussion with one of your colleagues who asks you what you thought of the ending and whether you had guessed who the killer was. To answer these questions, you need to remember the reflections you had while reading the book. These daily life situations illustrate that long-term memory is not only used to retain information about the external world but also internal mentation (note that here we use the terms *thought* and *internal mentation* interchangeably to refer to any kind of mental representation whose content does not represent current sensory input, irrespective of whether it is self-generated or triggered by the external environment). Memory for internal mentation (e.g., intentions, future plans, solutions to problems, impressions of others, interpretations of events, and so on) indeed plays a key role in guiding our decisions, actions, and social relations. Surprisingly, however, relatively little is known about the principles that govern memory for thoughts experienced during real-world events.

Research on the stream of thought in daily life has shown that the mean duration of a thought segment is about 14 seconds, suggesting that people may experience between 4,000 and 5,000 thoughts during a 16-hour day^1,2^. Although the ability to remember internal mentation has obvious benefits, it is likely that most thought segments are quickly forgotten, and that people are only able to remember a small portion of their daily thoughts at any given moment. Several studies have sought to identify thought characteristics that predict their probability of being retained in memory. In a precursor study, Brewer^3^ used an experience sampling procedure to assess memory for thoughts in daily life. Participants were asked to carry beepers that went off at random times during their daily activities and, each time they were probed, they had to write a short description of what they were thinking and to rate several features of their thoughts. After various retention intervals, participants were presented with their written descriptions and were asked to assess how well they remembered each thought. Brewer found that thoughts that received higher recall scores were rated as more emotionally pleasant, personally significant, and exciting during the experience sampling phase.

Memory for internal mentation has also been investigated in the context of reality monitoring—the processes involved in discriminating between memories for real and imagined events^4,5^. Johnson and colleagues^6^ asked participants to remember a real event (a social occasion, a trip to the library, or a visit to the dentist) and an imagined event (a dream, a fantasy, or an unfulfilled intention), and then to rate their memories on a wide range of characteristics (e.g., visual details, spatial and temporal information, emotional intensity). When comparing the two kinds of memories, it was found that memories for real events contained more perceptual details and contextual information, whereas memories for imagined events were more intense, seemed to have more implications at the time, and were thought about more often. Similar differences in the perceptual and contextual details of memories were observed when participants perceived or imagined a series of events in laboratory conditions (e.g., having a cup of coffee with some cookies), and the perceptual characteristics of memories decreased more over time for imagined than perceived events^7^.

A more recent line of research focused on memory for future-oriented thoughts (for a taxonomy of prospection, see ref.^8^). Studies of prospective memory investigate the ability to remember to perform intended actions^9,10^. While much research examined memory for intentions that were formed in the laboratory^11–13^, a few studies have assessed prospective memory in daily life. In particular, research using experience sampling methods demonstrated that prospective memory occupies our thoughts approximately 15% of the time^14,15^. Other studies investigated memory for the content of imagined future events^16^, what Ingvar^17^ referred to as “memory of the future.” In a series of experiments, Klein and colleagues found that evaluating a list of items when mentally simulating a future event (e.g., planning a camping trip) promotes memory for these items^18,19^, especially when the future simulation involves a personally familiar scenario^20^. McLelland and colleagues^21^ asked people to imagine a series of future events and to rate various characteristics of their mental simulations; memory for simulations was later assessed using a cued-recall test. Imagined events that were more detailed, more plausible, and more familiar were more likely to be remembered. Another study showed that imagined future events that could be recalled without the help of cues (i.e., in a free recall task) were rated as more personally important and involved more intense emotions^22^.

While these studies provide clues about the factors that influence memory for thoughts, little is known about the processes structuring the retrieval of internal mentation. Most notably, it remains unknown whether principles that govern the recall of stimuli from the external environment also apply to memory for thoughts. For instance, it is well established that the free recall of a list of items (e.g., words) demonstrates *primacy* and *recency* effects: people are more likely to recall items from the beginning and end of the list, resulting in a U-shaped curve of recall accuracy as a function of serial position^23,24^. Serial position within a list has also been found to influence the order in which items are retrieved (for reviews, see refs^25–27^). When a delay separates encoding from the free recall phase, individuals are more likely to initiate retrieval with one of the first items of the list^28^. Furthermore, a *temporal contiguity* effect with forward asymmetry is typically observed in free recall tasks: the order in which items are recalled tends to be similar to their original order of presentation (i.e., there is a strong tendency to transition between temporally contiguous items, with forward transitions being more likely than backward transitions)^29^. Taken together, these findings indicate that the serial position of items is a major predictor of recall likelihood and dynamics in episodic memory for external stimuli.

Although the lists of items that are used in typical laboratory experiments are inherently different from internal mentation, there are reasons to suspect that similar structuring effects might govern thought recall. Thinking is a serial process in which thoughts follow each other in a continuous flow that William James^30^ described as a “stream of thought”. People tend to divide this thought flow in individual segments that possess specific features and/or focus on distinct topics^2,31,32^. In other words, ongoing thought shifts from one unit to the next in a serial fashion (i.e., one usually experiences a single thought at a given time), akin to the serial presentation of stimuli in classical episodic memory experiments. Therefore, it could be that the effects of serial position that have been described in the free recall of external stimuli (i.e., primacy, recency, and contiguity effects) are also at play when people attempt to retrieve internal mentation.

An important difference between the stream of thought and lists of items in memory experiments is that thoughts tend to be organized in coherent sequences, whereas items in laboratory experiments are typically presented in random order (see ref.^33^ for a discussion of this point in relation to the retrieval of autobiographical events). In daily activities, thoughts may follow, at least in part, the structure of ongoing events. There is extensive evidence that people automatically segment everyday activities into events and sub-events according to changes in actions, contexts, and goals^34,35^. Transitions between events—referred to as *event boundaries*—are generally better remembered than event middles and determine the organization of episodic memories^36–38^. While these studies focused on memory for external stimuli, event boundaries might also serve as anchors that structure the retrieval of internal mentation. Thus, besides serial position effects, the retrieval of thoughts experienced during real-world events might also be influenced by event segmentation.

**Figure 1:**
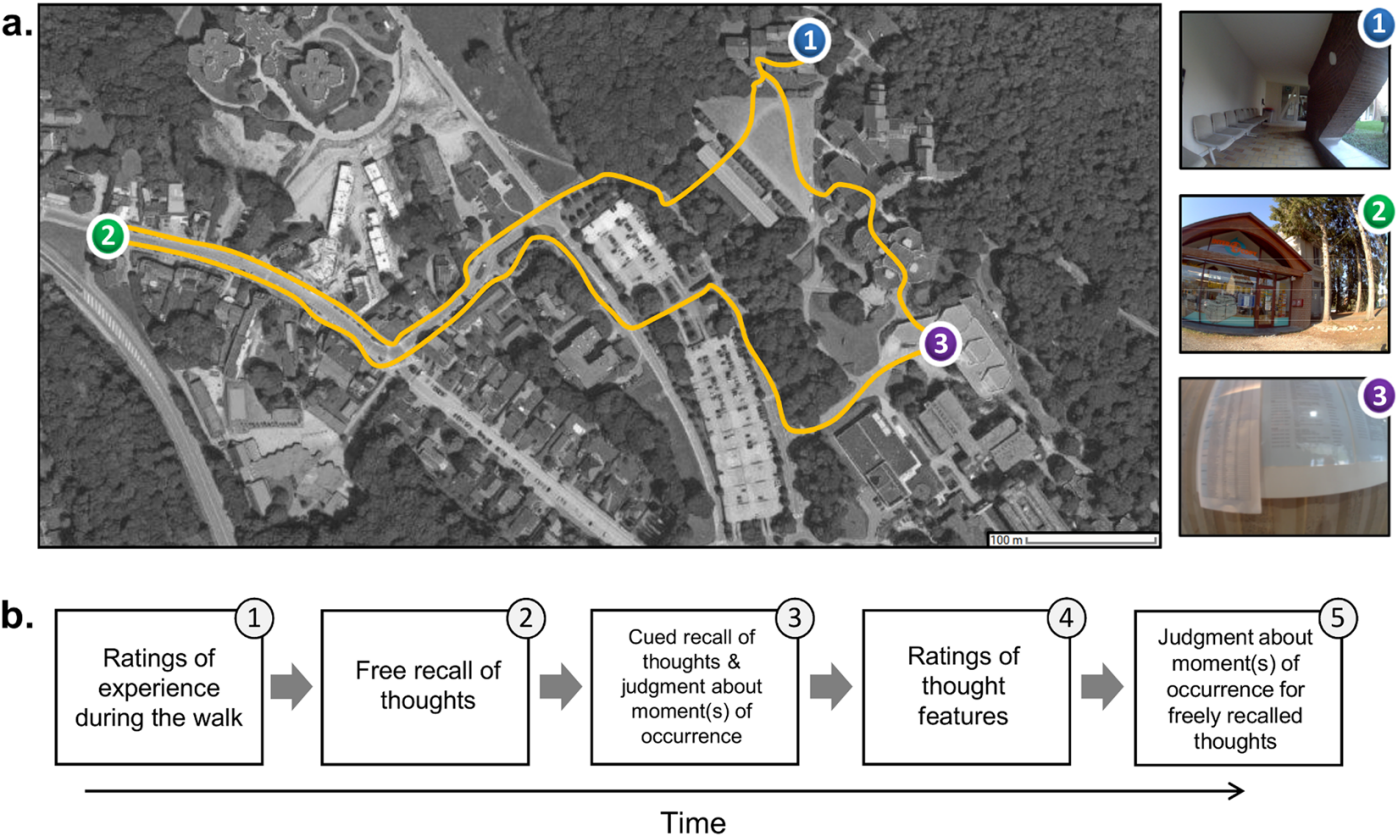
Illustration of the experimental procedure. Panel (a) illustrates the walk on the campus of the University of Liège (Belgium) and shows examples of pictures taken by the wearable camera. Participants had to (1) leave the testing room, (2) check the opening hours of a copy center, and (3) look on the door of a classroom to check the title of a lecture. Panel (b) illustrates the sequence of tasks participants received immediately after the walk. Participants had to (1) rate their experience during walk, (2) freely recall all the thoughts they experienced during the walk, (3) recall as many additional thoughts as they could while reviewing the picture of their walk and determine which picture(s) best illustrated the moment(s) of occurrence of each retrieved thoughts, (4) rate the phenomenological features of each retrieved thought from both recall phases, and (5) choose the picture(s) that best illustrated the moment(s) of occurrence for the thoughts they retrieved in the free recall task. The satellite imagery map used in the figure was created by the first author with Google Maps. Imagery ©2019 Aerodata International Surveys, DigitalGlobe, GeoContent, Map data ©2019 Google. All other photographs were taken by the first authors.

The main aim of the present study was to test these hypotheses on the structure of thought recall. To examine these questions, we developed a novel experimental paradigm that capitalizes on wearable camera technology^39,40^. Participants experienced a series of events during a walk on the university campus while wearing a wearable camera that automatically captured the content and timing of events. Then, they received a surprise free recall task in which they had to recall all the thoughts they experienced during the walk. The temporal structure of thought recall was investigated by later asking participants to determine when each recalled thought occurred during the walk by selecting the corresponding picture taken by the wearable camera. This allowed us to examine whether the retrieval of internal mentation is structured by the principles of primacy, recency, and temporal contiguity with forward asymmetry. In addition, the influence of event boundaries on thought recall was assessed by asking participants to perform two specific actions during the walk, which served as event boundaries (see Fig. 1).

Our second aim was to identify thought features that predict their accessibility in memory. To address this question, a cued recall task was presented following the free recall task, in which participants reviewed the pictures that had been taken during their walk and described all additional thoughts (i.e., not reported during free recall) that the pictures reminded them of; a previous study indeed showed that such pictures are particularly effective cues to help the retrieval of internal mentation^41^. Thoughts produced during the free recall and cued recall tasks were then rated on various dimensions (e.g., visual imagery, personal importance, affective valence; see Table 1), which allowed us to identify features that differentiated between thoughts that were accessible without the help of cues (i.e., thoughts produced in the free recall task) and thoughts that were only accessed when retrieval cues were presented (i.e., thoughts produced in the cued recall task).

**Table 1:** Thought Characteristic Questionnaire.

No.	Item	Response scale
1.	I could remember this thought without effort… (free recall only)	from 1 “*not at all*” to 7 “*completely*”
2.	During the walk this thought came to my mind…	1 “*involuntary*” to 7 “*deliberately*”
2a.	If this thought, came to my mind involuntary (rating <4 on item 2), it was triggered by… (allowed to pick several answers)	(A) The external environment
		(B) A physical sensation
		(C) A previous thought
		(D) No specific trigger
		(E) I can’t remember
3.	This thought was directly related to the walk…	1 “*not at all*” to 7 “*completely*”
4.	This thought was about something that I was directly perceiving/feeling…	1 “*not at all*” to 7 “*completely*”
5.	This thought was in the form of visual images…	1 “*not at all*” to 7 “*completely*”
6.	This thought was in the form of inner speech…	1 “*not at all*” to 7 “*completely*”
7.	This thought was about something… (allowed to pick several answers)	(A) Prior to the present moment
		(B) In the present moment
		(C) After the present moment
		(D) Not related to a specific moment
7a.	If this thought was about something prior/after the present moment, what was its temporal distance… (give a numbered approximation)	… s … min … hour … day … month … year
8.	The affective content of this thought was…	−3 “*very negative*” to +3 “*very positive*”
9.	This thought came back to my mind at different times during the walk…	1 “*not at all*” to 7 “*very often*”
10.	This thought comes back to my mind in daily life…	1 “*not at all*” to 7 “*very often*”
11.	This thought was about something important for me…	1 “*not at all*” to 7 “*completely*”
12.	This thought was about something…	1 “*vague/general*” to 7 “*concrete/specific*”
13.	This thought was about me…	1 “*not at all*” to 7 “*completely*”
14.	This thought was about others…	1 “*not at all*” to 7 “*completely*”
15.	This thought was about something…	1 “*usual*” to 7 “*unusual*”
16.	I had this thought in mind for…	1 “a *very short amount to time*” to 7 “a *long time*”
17.	I tried to suppress this thought from my mind…	1 “*not at all*” to 7 “*completely*”
18.	The main function of this thoughts was (allowed to pick several answers)	(A) Take a decision/solve an issue
		(B) Planning
		(C) (Re)evaluate a situation
		(D) Self-entertainment/feeling better
		(E) Other
		(F) No apparent function

Considering that an adaptive function of episodic memory is to provide information that can be used to support future decisions and behavior^42–45^, we expected that the future orientation and planning function of thoughts would predict their accessibility in memory. Furthermore, we predicted that features that are well-known to influence memory for external stimuli, such as self-relevance^46^, distinctiveness^47^, emotional valence^48^, and rehearsal^7,49,50^ would also predict the accessibility of thoughts in memory. Finally, recent studies on mind-wandering have shown that stimulus-dependence and task-relatedness are important dimensions of thoughts^51–53^, with people spending from 30% to 50% of their daily life engaged in mind-wandering episodes that are unrelated to their ongoing activity and decoupled from current sensory input^54,55^. At a more exploratory level, we investigated whether these two dimensions predict the accessibility of thoughts in memory.

## Results

### Frequency and temporal distribution of memories for thoughts

In total, the 44 participants reported memories for 819 thoughts, with a mean of 18.61 thoughts per participant (95% CI [16.24, 20.99]). The mean number of thoughts reported in the free (*M = *9.59, 95% CI [8.43, 10.76]) and cued (*M* = 9.02, 95% CI [7.22, 10.83]) recall phases was equivalent [*t*(43) = 0.61, *p* = 0.55, Cohen’s *d* = 0.09]. The majority of thoughts reported in the free recall phase were judged to have occurred at a single moment during the walk (406 out of 422 thoughts or 96.2%). Of the remaining thoughts, four occurred twice during the walk, eleven were recurrent, and one could not be associated with a precise time of occurrence because the participant did not remember exactly when it happened. In the cued recall phase, 394 of the 397 reported thoughts (99.2%) were associated with a single time of occurrence (the remaining three thoughts were associated with two distinct moments of occurrence).

**Figure 2:**
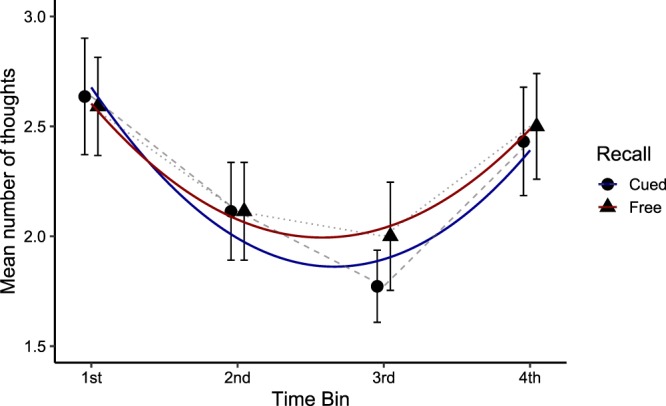
Distribution of retrieved thoughts for each time bin of the walk. Colored lines represent the fitted quadratic term. Error bars represent the standard error of the mean.

To investigate whether thought recall showed primacy and recency effects, we divided the walk of each participant in 4 time bins of equal duration and calculated the number of thoughts reported in the free and cued recall tasks for each time bin. Then, we performed a mixed-effects growth curve analysis with higher-order polynomials to assess the shape of the serial position effect (see the Methods section for details). Compared to a baseline (random intercept only) model, the use of a first-order polynomial (linear term) to model change in thought recall across time bins did not improve model fit [χ²(1) = 1.02, *p* = 0.31]. On the other hand, the use of a second-order polynomial (quadratic term) improved model fit [χ²(1) = 13.60, *p* < 0.001], with the shape of the serial position curve showing clear primacy and recency effects (Fig. 2). Adding the effect of recall type [χ²(1) = 0.07, *p* = 0.79] and the interactions between recall type and the polynomial terms did not improve model fit [χ²(1) = 0.19, *p* = 0.66, for the interaction with the linear term; χ²(1) = 0.13, *p* = 0.72, for the interaction with the quadratic term], indicating that the primacy and recent effects did not differ between the free and cued recall phases. The coefficients, standard errors, *t* and *p*-values for the fixed effects of the optimal (quadratic) model are presented in Table 2.

**Table 2:** Fixed effects of the optimal (quadratic) model for the growth curve analysis on the number of retrieved thoughts over the 4 time bins of the walk.

	*b*	*SE*	*df*	*t*	*p*-value
Intercept	2.27	0.15	44	15.61	<0.001
Linear term	−0.15	0.14	264	−1.04	0.30
Quadratic term	0.54	0.14	264	3.74	<0.001

**Figure 3:**
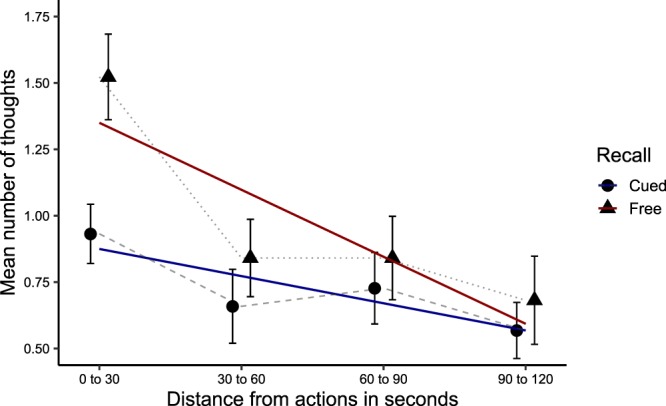
Distribution of recalled thoughts according to temporal distance from the actions (event boundaries). Colored lines represent the fitted linear term. Error bars represent the standard error of the mean.

To investigate the influence of event boundaries on thought recall, we examined the number of retrieved thoughts as a function of their temporal proximity to the actions defining event boundaries. For the 4 minutes surrounding each action, we computed the numbers of recalled thoughts aggregated in four 1-minute time bins (i.e., 30 s before/after the action; 30 to 60 s before/after the action; 60 to 90 s before/after the action; and 90 to 120 s before/after the action; see Fig. 3 for the mean number of thoughts recalled for each time bin). A growth curve analysis showed a significant linear relationship between thought recall and temporal distance from actions [χ²(1) = 14.89, *p* < 0.001], indicating that more thoughts were recalled in proximity to actions; adding the quadratic term did not improve model fit [χ²(1) = 2.47, *p* = 0.12]. Adding the effect of recall tasks improved the model fit [χ²(1) = 6.18, *p* = 0.01], indicating that more thoughts were retrieved in the free recall task than in the cued recall task. Although the effect of temporal proximity seemed slightly more pronounced in the free recall task (see Fig. 3), the interaction between the recall tasks and the linear term did not improve model fit [χ²(1) = 2.82, *p* = 0.09], nor did the interaction between the recall tasks and the quadratic term [χ²(1) = 1.06, *p* = 0.30]. The coefficients, standard errors, *t* and *p*-values for the fixed effects of the optimal (linear) model are presented in Table 3. Overall, these results indicate that the number of recalled thoughts increased in proximity to event boundaries. Further analyses showed that this effect was similar for time bins preceding and following event boundaries (see Section 2 of the Supplementary Information).

**Table 3:** Fixed effects of the optimal (linear) model for the influence of the temporal proximity to actions on the number of retrieved thoughts.

	*b*	*SE*	*df*	*t*	*p*-value
Intercept	0.72	0.09	98.25	8.42	<0.001
Linear term	0.40	0.10	308.00	3.94	<0.001
Recall type	0.25	0.10	308.00	2.49	0.01

To investigate the temporal structure of free recall protocols, we examined how participants initiated the recall of their thoughts. The first thought (with a single time of occurrence) produced in the free recall task occurred, on average, 2.58 min (95% CI [1.29, 3.87]) after the start of the walk, indicating a primacy effect in the way retrieval was initiated (considering that the walk lasted around 25 minutes). Next, we examined to what extent the order of recalled thoughts corresponded to the order of thought occurrence during the walk (see the Methods section for details on this analysis). This showed that, on average, participants recalled 83% of thoughts following a chronological order (95% CI [77, 88]), indicating a strong temporal contiguity effect with forward asymmetry. All but two participants reported a majority of thoughts following a chronological order, suggesting that this effect was ubiquitous among participants. Unsurprisingly given that participants started reviewing the pictures of their walk from the beginning, 95% of thoughts reported during the cued recall task followed a chronological order (95% CI [88, 100]). The first thought produced in the cued recall task occurred, on average, 2.98 min after the start of the walk (95% CI [1.30, 4.67]).

### Characteristics of recalled thoughts

Our next goal was to compare the characteristics of thoughts produced in the free and cued recall tasks to identify thought features that were related to their accessibility in memory (i.e., thought features that differentiated between thoughts that were remembered without the help of the pictures and thoughts that came to mind when reviewing the pictures). Mean ratings and 95% CI for each thought dimension are presented in Table 4 for the free and cued recall tasks. Paired *t*-tests showed that the thoughts produced in the free recall phase were about more important and unusual topics, were more frequently in mind, and were associated with more suppression attempts than the thoughts recalled in the cued recall phase. For other characteristics, the average ratings indicated that both kinds of thoughts were mostly involuntary, stimulus-dependent, specific, and affectively neutral (Table 4).

**Table 4:** Mean ratings for the characteristics of retrieved thoughts and two-tailed paired t-tests on differences between free vs cued recall.

Dimension	Free vs. cued recall				
	Free recall Mean [95% CI]	Cued Recall Mean [95% CI]	*t(43)*	*p*	*Cohen’s d*
1. Deliberate	2.88 [2.51, 3.25]	2.64 [2.23, 3.06]	1.32	0.19	0.20
2. Walk-related	3.86 [3.52, 4.21]	3.84 [3.42, 4.26]	0.13	0.90	0.02
3. Stimulus-dependence	4.66 [4.35, 4.96]	4.76 [4.40, 5.12]	−0.62	0.54	0.09
4. Visual format	4.13 [3.76, 4.51]	4.00 [3.52, 4.48]	0.85	0.40	0.13
5. Inner speech format	4.58 [4.21, 4.96]	4.36 [3.93, 4.79]	1.32	0.19	0.20
6. Affective valence	0.18 [0.03, 0.33]	0.22 [0.04, 0.40]	−0.40	0.69	0.06
7. Life frequency	2.83 [2.57, 3.08]	2.61 [2.26, 2.95]	1.51	0.14	0.23
8. Personal importance	2.55 [2.28, 2.81]	2.23 [1.91, 2.55]	**2.44**	**0.02**	**0.37**
9. Specific/concrete	5.00 [4.63, 5.38]	4.71 [4.28, 5.15]	1.88	0.07	0.28
10. Self-related	3.86 [3.54, 4.17]	3.65 [2.22, 4.07]	0.96	0.34	0.14
11. Other related	3.75 [3.44, 4.07]	3.65 [3.37, 3.92]	0.59	0.56	0.09
12. Unusual	3.17 [2.85, 3.49]	2.75 [2.36, 3.13]	**2.60**	**0.01**	**0.39**
13. Suppression	1.80 [1.57, 2.04]	1.57 [1.33, 1.81]	**2.83**	**0.01**	**0.43**
14. Walk Freq./Time in mind	3.26 [2.99, 3.52]	2.39 [2.12, 2.65]	**7.06**	<**0.01**	**1.06**

Note: Each dimension ranged from 1 to 7, except for the affective valence dimension the range of which went from −3 to +3.

**Figure 4:**
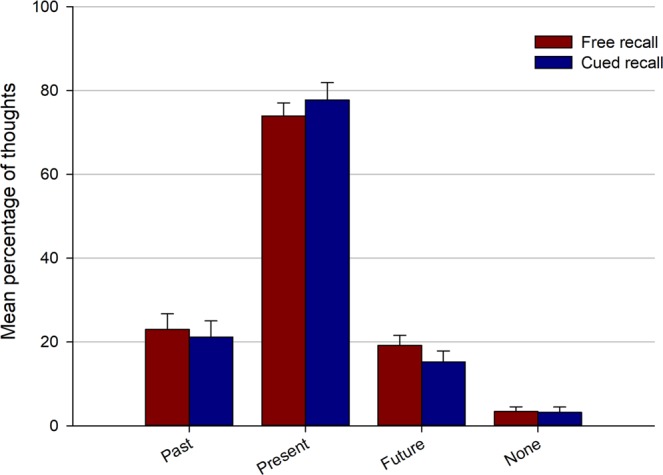
Temporal orientation of thoughts retrieved in the free and cued recall phases. Error bars represent the standard error of the mean.

The temporal orientation of retrieved thoughts is presented in Fig. 4. A 2 (recall type: free vs. cued) × 4 (orientation: past vs. present vs. future vs. none) repeated measures ANOVA showed a main effect of orientation [*F*(3, 129) = 118.36, *p* < 0.001, ${\eta }_{p}^{2}$ = 0.73], but no main effect of recall type [*F*(1, 43) = 1.3, *p* = 0.30, ${\eta }_{p}^{2}$ = 0.02] and no interaction [*F*(3, 129) = 1.28, *p* = 0.29, ${\eta }_{p}^{2}$ = 0.03]. Post-hoc tests (Tukey’s HSD) showed that thoughts about the present were the most frequent (all *p*s < 0.001), whereas thoughts without temporal orientation were the least frequent (all *p*s < 0.001); there was no difference between past- and future-oriented thoughts (*p* = 0.64). The number of future-oriented thoughts reported in the two recall tasks did not differ across the four time bins of the walk (see Section 3, Fig. S3 of the Supplementary Information). The proportion of past and future thoughts that were specifically located in time was 89% (95% CI [81, 98]) and 84% (95% CI = [75, 94]), respectively. However, there was an important variability in temporal distance across thoughts and given that only seven participants reported past and future thoughts with a specific temporal location in both recall tasks, we did not conduct further analyses on this dimension.

**Figure 5:**
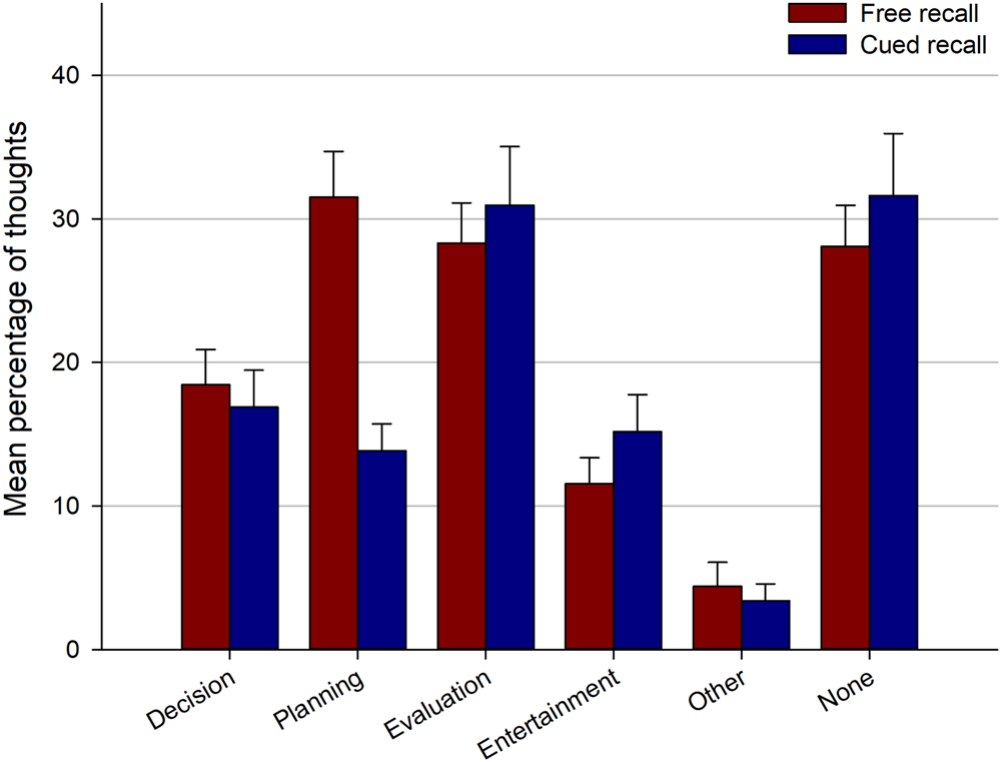
Functions attributed to thoughts retrieved in the free and cued recall phases. Error bars represent the standard error of the mean.

The perceived function(s) of retrieved thoughts are presented in Fig. 5. A 2 (recall type: free vs. cued) × 6 (function: decision vs. planning vs. evaluation vs. self-entertainment vs. other vs. none) repeated measures ANOVA showed a main effect of recall type [*F*(1, 43) = 6.7, *p* = 0.01, ${\eta }_{p}^{2}$ = 0.13], a main effect of function [*F*(5, 215) = 16.99, *p* < 0.001, ${\eta }_{p}^{2}$ = 0.28], and a significant interaction [*F*(5, 215) = 6.05, *p* < 0.001, ${\eta }_{p}^{2}$ = 0.12]. Post-hoc tests (Tukey’s HSD) indicated that the proportion of thoughts that fulfilled a planning function was higher in the free than cued recall task (*p* < 0.001), whereas there was no difference between the two recall tasks for any of the other functions (all *p*s > 0.90). The increased number of planning-related thoughts in the free recall task was constant across the four time bins of the walk (see Section 3, Fig. S4 and Table S3 of the Supplementary Information). Given this increased accessibility of thoughts that fulfilled a planning function, we further examined thought features that differentiated between planning- vs. non-planning-related thoughts. Results showed that thoughts involving planning processes were more specific, deliberate, self-related, recurrent (both during the walk and in the participant’s life), and related to the walk than thoughts that did not involve planning (see Section 4 of the Supplementary Information).

**Figure 6:**
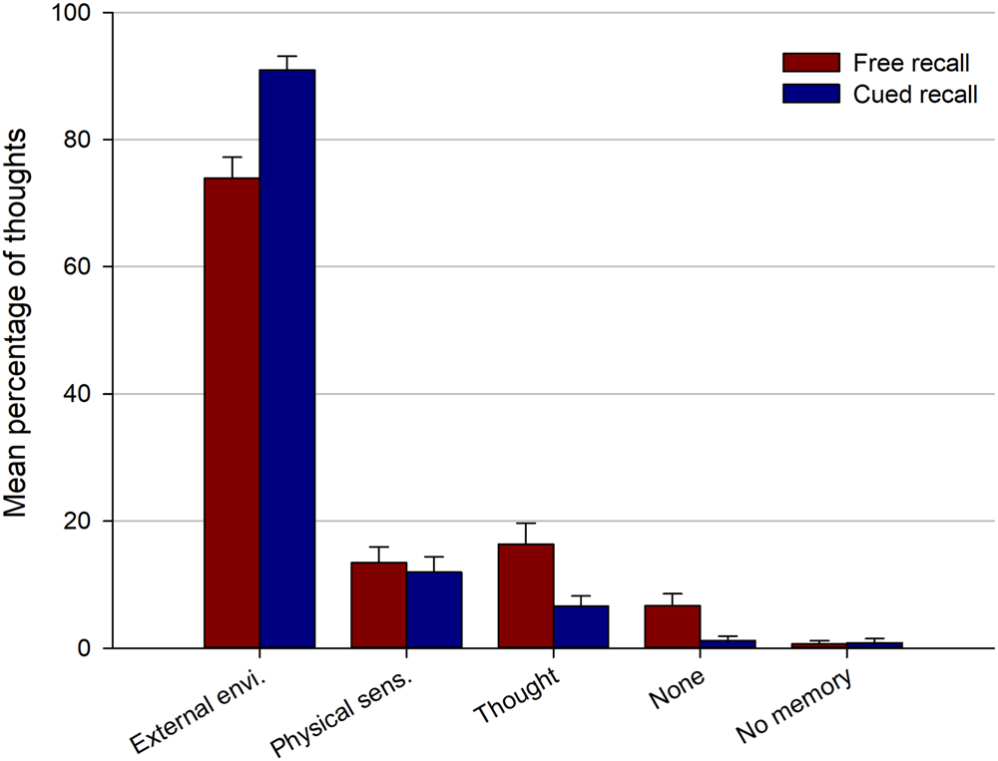
Triggers of thoughts that occurred spontaneously during the walk. Error bars represent the standard error of the mean.

Finally, we examined the triggers of the subset of retrieved thoughts that were rated as having occurred involuntarily during the walk (Fig. 6). Three participants were excluded from this analysis because they did not report any involuntary thoughts in the cued recall task. The category “no memory” was also excluded (i.e., trials for which participants did not remember how the thought was triggered) because it was only used four times across the two recall tasks. A 2 (recall type: free vs. cued) × 4 (trigger: environment vs. sensation vs thought vs. none) repeated measures ANOVA showed no main effect of recall type [*F*(1, 40) = 0.13, *p* = 0.91, ${\eta }_{p}^{2}$ < 0.001] but a main effect of trigger [*F*(3, 120) = 295.81, *p* < 0.001, ${\eta }_{p}^{2}$ = 0.88] and interaction [*F*(3, 120) = 13.88, *p* < 0.001, ${\eta }_{p}^{2}$ = 0.26]. Post-hoc tests (Tukey’s HSD) showed that the external environment was the most frequent trigger of involuntary thoughts for both recall tasks and was more frequent for cued than freely recalled thoughts (all *p*s < 0.001). Involuntary thoughts were more frequently triggered by preceding thoughts in the free than cued recall task (*p* = 0.05). There was no difference between the two recall tasks for thoughts cued by physical sensations and thoughts with no identifiable trigger (both *p*s > 0.61).

For completeness, we also examined whether the characteristics of retrieved thoughts varied depending on their moment of occurrence during the walk; these additional analyses showed that serial position and event boundaries had relatively little influence on the characteristics of recalled thoughts (see Sections 5 and 6 of the Supplementary Information).

## Discussion

While memory for internal mentation (e.g., future plans, solutions to problems, impressions of others) plays a key role in guiding decisions and actions, surprisingly little is known about the principles that govern memory for thoughts experienced during real-world events. The first aim of this study was to investigate whether the recall dynamics of internal mentation is structured by the principles of primacy, recency, and temporal contiguity, and is influenced by event boundaries. Our second aim was to identify thought features that predict their accessibility in memory. To investigate these questions, we asked participants to undertake a 25-min walk on the university campus while the content and timing of experienced events were recorded by a wearable camera. Participants then received free and cued recall tasks about the thoughts experienced during the walk and had to rate various features of recalled thoughts. The pictures taken by the wearable camera were used to estimate when recalled thoughts occurred during the walk, which allowed us to examine the structure of thought recall.

On average, participants retrieved 18.61 thoughts across the two recall tasks. Based on previous findings^2^ that thought segments last on average 14 s, this suggests that they only remembered between 15% and 20% of the thoughts they experienced during the walk. An important question, then, is whether memory for thoughts is governed by similar principles as memory for external information. Given that the stream of thought transitions from one thought segment to the next in a serial manner^2,30,32^, we predicted that the serial position of thoughts during the walk would affect their subsequent retrieval. In line with this hypothesis, we found that participants tended to initiate recall with a thought that occurred at the beginning of the walk, demonstrating a primacy effect in recall initiation that is similar to memory retrieval for external stimuli following a delay^28^. Furthermore, primacy and recency effects were observed in the frequency of thought recall, with fewer thoughts being recalled from the middle compared to the beginning and end of the walk (see Fig. 2), which again mimics the serial position effects observed in memory tasks for external stimuli^23,24^. Thought recall also demonstrated a clear temporal contiguity effect with forward asymmetry^29^, such that the order in which thoughts were recalled tended to be similar to their order of occurrence during the walk. Finally, besides serial position effects, our results suggest that event boundaries structured thought recall; more thoughts were retrieved in temporal proximity to event boundaries (see Fig. 3), as is also the case for external stimuli^34,36,37^.

The human memory system associates each new experience within a spatiotemporal context. There is evidence that this context is reinstated when the experience is recalled^56–58^, and serves as a cue to guide the retrieval of other associated experiences^59^. The role of context in memory recall accounts for temporal contiguity effects and may also explain primacy and recency effects, considering that the beginning and end of a list are contextually more distinct than the middle of the list, which facilitates the retrieval of associated items^60^. The present results suggest that this context model of memory search may also account for the structure of thought recall. When attempting to recall the thoughts they experienced during a given event, people may initiate memory search at the beginning of the event because it represents a particularly distinctive moment of experience and constitutes an event boundary^61,62^ that is easily accessed. In line with this proposal, we found that recalled thoughts that occurred near the beginning of the walk were rated as more easily retrieved (see Section 7 of the Supplementary Information). People may then use the episodic context reinstated with each retrieved thought to guide the retrieval of the next thought they experienced, such that the order of thought recall tends to approximate their initial order of occurrence. Thus, the retrieval of internal mentation and stimuli from the external world appear to be affected and structured (at least in part) by similar cognitive processes.

Interestingly, these serial position effects in thought recall were of similar magnitude in the free and cued recall tasks. A possible explanation for this finding is that the free recall of thoughts was guided by the mental replay of the walk, as was presumably the case in the cued recall task. In particular, participants may have used spatial representations as contextual anchors to guide the retrieval process. There is evidence that the recall of objects that have been learned in particular spatial locations demonstrates spatial clustering (i.e., people tend to transition between items that have been learned at nearby spatial locations), in addition to temporal contiguity effects, suggesting that both spatial and temporal contextual features are used as cues during free recall^63^. In the same vein, people may use representations of spatial locations (in addition to serial position) to retrieve associated internal mentation. This recall strategy may be especially useful for involuntary thoughts, given that most of these were triggered by the external environment (see Fig. 6). In the present study, spatial and temporal contiguity were highly correlated, preventing us to analyze the specific effect of each contextual dimension. The roles of spatial and temporal information in the organization of thought recall could be disentangled in future studies by asking participants to use the same routes at different times during the walk.

Beyond spatial and temporal contiguity, the present results suggest that the retrieval of internal mentation is influenced by the general structure of associated events. People segment the continuous flow of experience into events and sub-events following changes in various dimensions of experience, including actions, contexts, and goals^34,35^. This segmentation process plays a key role in the organization of episodic memories and a well-established finding is that information close to event boundaries is better remembered than information at event middles^36–38^. While these studies focused on memory for external stimuli, the present results show that the retrieval of internal mentation was also influenced by event boundaries (defined by specific actions that participants had to perform during the walk). These results suggest that event boundaries may serve as anchors during memory search, thereby increasing access to associated thoughts.

The second aim of this study was to identity thought dimensions that predict their accessibility in memory. An important finding here is that twice as many thoughts judged as fulfilling a planning function were retrieved in the free compared to cued recall task (see Fig. 5). These results are concordant with the proposal that an important function of episodic memory is to store information that is useful to guide decisions and future behavior^16,42–45,64^. They also fit well with the idea that the greater accessibility of planning thoughts in memory results from an evolutionary process whereby mental contents that enhance survival chances are better remembered^18–20^. Our results extend previous findings by showing that this greater accessibility of planning thoughts in memory not only occurs in laboratory tasks but also for internal mentation generated in a real-world setting. It should be noted, however, that many planning thoughts recalled in the present study pertained to ongoing events (rather than more distant future events); indeed, recalled thoughts were predominantly rated as present-oriented (see Fig. 4). Finally, it is also worth noting that planning thoughts might entail a range of cognitive processes that could increase their accessibility in memory. For instance, several studies that examined the features of planning vs. non-planning thoughts found that the former are more elaborate, require more cognitive effort^31,65,66^, and are more tightly linked with the self and personal goals^52,67^. These differences in thought features were mostly replicated in the present study (see Section 4 of the Supplementary Information) and might explain the enhanced accessibility of thought involving planning processes.

Besides planning, we found that thoughts that were more accessible in memory were judged to have occurred more often and to have stayed a longer time in mind (see Table 4), a finding concordant with previous studies on rehearsal^7,49,50^. Also in line with previous findings^3,22^, thoughts that were recalled without the help of cues were rated as more personally important. A possible explanation for this finding is that important thoughts are better integrated with higher-order autobiographical knowledge structures (notably, personal goals), resulting in a more efficient encoding and increased accessibility in memory (for a similar proposal regarding the perceptual aspects of autobiographical memories, see ref.^68^). A third finding was that thoughts that were more accessible were rated as being about more unusual topics. Unusual topics may stand out among the stream of thought and lead to better memory, akin to the novelty^69,70^ and distinctiveness^47^ effects found in laboratory memory tasks. Finally, we also found that thoughts that participants attempted to suppress were subsequently more accessible in memory. Although one may have expected that thought suppression would reduce memory accessibility in a similar way as directed forgetting^71^, it could be that the cognitive effort and attention given to thoughts when attempting to suppress them led to a stronger encoding of their content and features. Future studies should be conducted to formally assess these proposals.

While the present study represents a first attempt to characterize the recall dynamics of internal mentation, a limitation of our paradigm is that we have no record of the thoughts that were experienced during the walk. Our analyses of recall dynamics assume that the occurrence of thoughts was equally distributed across the walk, such that differences in the frequency of recalled thoughts as a function of time (e.g., primacy and recency effects) would reflect the organization of memory retrieval. However, it remains possible that the observed effects result in part from differences in thought distribution at encoding. To address this issue, future studies could attempt to monitor the occurrence of thoughts during the walk, for instance using experience sampling methods^3,72^. A limitation of this procedure, however, is that the recording of experienced thoughts would likely influence memory encoding. Thus, one may need to combine multiple, complementary methods to shed further light on the respective roles of encoding and retrieval processes in memory for internal mentation.

In conclusion, this study provides an initial attempt to lift the veil on the processes that structure the recall of internal mentation. We introduced a novel experimental paradigm to investigate memory for thoughts experienced during real-world events and found that the dynamics of thought recall showed primacy, recency, and temporal contiguity effects and was influenced by event boundaries. Furthermore, the accessibility of thoughts in memory was related to several factors and notably their involvement in planning processes. Together, these results shed new light on the principles that govern memory retrieval for internal mentation and suggest that at least partially similar processes structure the retrieval of thoughts and stimuli from the external environment.

## Methods

The fully anonymized data files and coded data can be obtained upon request to the first author. The research reported here was approved by the Ethics Committee of the Department of Psychology, Speech Therapy, and Education of the University of Liège. Informed consent was obtained from all the participants before the beginning of the study and the methods were carried out in accordance with the relevant guidelines and regulations. We report how we determined our sample size, all data exclusions, all manipulations, and all measures below^73^.

### Participants

Forty-four participants (25 women) took part in this study (mean age = 22.52 years, *SD* = 3.41; range = 19–36 years). All of them reported to be familiar with the campus of the University of Liège where the study took place. Three additional participants were tested but not included in the final sample because of a malfunction of the wearable camera (one participant) or because they did not comply with the instructions (two participants). The sample size was determined a priori^74^ in order to have a statistical power of 90% (alpha value of 0.05) to detect medium-sized effects in within-subject analyses. None of the participants reported a history of neurological or psychiatric disorder or the use of medication that could affect their cognitive functioning.

### Materials and procedure

#### Walk on campus phase

Participants undertook a walk on the campus of the University of Liège while wearing an Autographer (OMG Life Ltd), a lifelogging camera that automatically, silently, and continuously takes pictures from the first person perspective^36,41^ (for an illustration of the experimental procedure, see Fig. 1**)**. More specifically, they were instructed to go to different locations and to perform a series of actions that simulate daily life activities: they first had to go to a local copy center to check opening hours, then went to a specific building to check the title of the lecture being given in a classroom, and finally came back to the testing room. Participants could take the route they wanted to go from one location to the other and they were asked to behave normally (i.e., as they would in their daily life), except that they should not use their smartphone, listen to music, or engage in discussions with people along the walk. On average, the length of the walk was 24.92 min (95% CI [24.24, 25.60]). Pictures were taken by the Autographer every 7.02 s on average (95% CI [6.9, 7.14]), and the mean total number of pictures taken per participant was 213.61 (95% CI [206.57, 220.66]). Importantly, participants were not informed that they would later be asked to remember the thoughts they experienced during the walk. As a cover story, they were told that they were taking part in a pilot study, the purpose of which was to test the quality of pictures taken by the Autographer in order to determine whether this device would be suitable for subsequent studies in which people would wear it in their daily life.

Immediately after coming back to the testing room, participants were asked to rate to what extent they were attentive to the external environment during the walk, to what extent they experienced thoughts unrelated to the present situation, like remembrances of past events or thoughts about future events (i.e., mind-wandering), and to what extent they behaved naturally. The three ratings were made on a seven-point Likert scale ranging from 1 (*not at all*) to 7 (*totally)*. Mean ratings were respectively 4.65 (95% CI [4.34, 4.96]), 4.74 (95% CI [4.30, 5.19]), and 5.56 (95% CI [5.22, 5.90]), indicating that, on average, participants were moderately attentive to the external environment, experienced some mind-wandering, and behaved naturally.

#### Thought recall phase

After rating their experience, participants were asked to recall out loud all thoughts, ideas, reflections, and any other mentation that they experienced during the walk. They were instructed to report these thoughts as they came to mind (no retrieval strategy was suggested by the experimenter) and to describe their memories in as much detail as possible. However, if they felt uncomfortable with disclosing the content of some thoughts, they could describe them using a few general keywords. All responses were audio-recorded. On average, participants started their recall 6.13 min after the end of the walk, 95% CI [5.72, 6.54].

After this free recall task, participants received a cued recall task. Specifically, they were asked to review all the pictures taken by the Autographer and to describe all additional thoughts (i.e., thoughts not retrieved during the free recall task) that the pictures reminded them of. All the pictures were reviewed in a chronological order, at the participants’ own pace, but participants could go backward if they wished by using the left and right arrow keys on the keyboard. Participants were asked to verbally describe any additional thought they retrieved (all responses were audio-recorded) and to choose the photograph(s) that best corresponded to the moment when they experienced the reported thought. The corresponding filename was recorded in order to determine when exactly each remembered thought occurred during the walk. If participants were unsure whether or not a thought had already been recalled in the free recall task, they were instructed to report this thought.

Following the two recall tasks, participants listened to the recording of each recall phase and were asked to rate various characteristics of each reported thought (see Table 1 for a description of each item) while the experimenter made a written transcription of all thoughts. When participants reported having experienced the exact same thought at different times during the walk, this thought was rated only once. Thoughts from the free recall phase that were inadvertently recalled a second time in the cued recall phase were not rated again and were discarded from all subsequent analyses.

Finally, participants reviewed the pictures of their walk a second time and, with the help of the written transcriptions of thoughts reported in the free recall task, they were asked to choose the picture that best corresponded to the moment when they experienced each thought. At the end of the testing session, participants were debriefed and asked whether they had guessed that memory for thoughts would be tested. None of them reported having guessed the purpose of the study.

#### Data preparation for statistical analyses

To examine the presence of primacy and recency effects on the number of thoughts recalled, we first determined the duration of the walk for each participant based on the shooting times of the first and last picture taken by the camera (i.e., when leaving and coming back to the testing room). We then divided this time interval into four bins of equal duration and computed for each participant the number of thoughts with a single occurrence that occurred in each time bin. Mixed-effects growth curve analysis^75,76^ was used to analyze changes in the number of thoughts recalled over the 4 time bins. The frequency of thought recall over time was modeled with second-order orthogonal polynomials (i.e., linear and quadratic terms) and fixed effects of the type of recall task (free vs. cued) on all time terms. The cued recall condition was treated as the baseline and parameters were estimated for the free recall condition. The models also included random effects of participants and participants by recall type variability. We first fitted a baseline (random intercept only) model and then the fixed effects of time terms and recall task (and their interaction) were added individually and their effects on model fit were evaluated using model comparisons. Improvements in model fit were evaluated using −2 times the change in log-likelihood, which is distributed as χ² with degrees of freedom equal to the number of parameters added. All analyses were carried out in R using the lme4 package^77^ and parameters were tested for significance with the lmerTest package^78^. Similar growth curve analyses were performed to investigate the effect of event boundaries (actions) on the number of recalled thoughts, with the difference that data were aggregated in 4 time bins depending on their temporal distance from the boundaries (i.e., less than 30 s, from 30 to 60 s, from 60 to 90 s, and from 90 to 120 s). Only participants were included as random effect in this latter model because the inclusion of participants by recall type variability in the random effects structure showed a singular model fit.

To examine whether thought recall demonstrates a temporal contiguity effect with forward asymmetry, we determined the time when each thought occurred by extracting temporal information associated with the corresponding picture (i.e., the time when the picture was taken). This allowed us to determine when each remembered thought occurred during the walk and the proportion of recalled thoughts that followed a chronological order. Specifically, we examined the succession of recalled thoughts two by two and computed the percentage of successive thoughts that followed a chronological order (e.g., if a participant reported thoughts A, B, C, and D in that order in the free recall task, we examined if A was experienced before B, B before C, and C before D during the walk; only thoughts that were associated with a single moment of occurrence were included in this analysis).

Finally, to determine whether the rating data from the Likert scales could be reduced for further analyses comparing thought features between the free and cued recall tasks, we examined relationships between ratings of the 15 thought features (item 1 was not included as it was only used in the free recall task). The multi-level correlation matrix (with the 819 thoughts nested within the 44 participants) of thought characteristics showed that only three correlation coefficients were larger than 0.30 (Table S8**)**, which is far below the recommendation for performing multivariate data reduction^79^. Thus, we examined each item separately in subsequent analyses, except that the ratings of frequency and time in mind (items 9 and 16 in Table 1) were pooled together given their high correlation (*r* = 0.61) and conceptual similarity. The two other coefficients with values above 0.30 revealed that there was a negative correlation (*r* = −0.39) between inner speech and visual images (indicating that thoughts tended to involve either inner speech or visual imagery) and a positive correlation between the personal importance of thoughts and their frequency of occurrence in daily life (*r* = 0.42).

## Supplementary information

Suplementary Information
