# Supplementary material for: The dynamics of memory retrieval for internal mentation
Source: Sci Rep. 2019 Sep 26;9:13927. doi: 10.1038/s41598-019-50439-y (PMC6763453; doi:10.1038/s41598-019-50439-y)
Supplement: Supplementary file 1 — Suplementary Information [file 41598_2019_50439_MOESM1_ESM.pdf]

Supplementary information

The dynamics of memory retrieval for internal mentation

David Stawarczyk[1*], Arnaud D'Argembeau[1,2]

[1] Department of Psychology, Psychology and Neuroscience of Cognition Research Unit, University of Liège, 4000 Liège, Belgium.

[2] GIGA-CRC In Vivo Imaging, University of Liège, 4000 Liège, Belgium.

Correspondence concerning this article should be addressed to David Stawarczyk, Department of Psychology, University of Liège, Quartier Agora, place des Orateurs 1, 4000 Liège 1, Belgium.

E-mail: d.stawarczyk@uliege.be. Tel.: 0032 4 3665979.

1. Number of retrieved thoughts as a function of spatial segments

In the main text, we report an analysis of the frequency of thoughts recalled as a function of 4 time bins of equal duration. We chose to divide the walk in 4 time bins in order to disentangle the effect of time from the structure of the walk in three segments (determined by the two actions performed during the walk; see **Figure 1**). Here, we aimed to further investigate how a model based on event boundaries (the two actions) fitted the data compared to the previous model based on time bins. To do so, the route was divided in three spatial segments based on the two actions (here we analyzed the number of thoughts recalled per minute of the walk rather than raw numbers of thoughts to take into account the fact that the three segments had different time lengths) and we compared the amount of variance explained by a model with these three segments compared to the time bins model. R^2 were computed in R for each model using the Mumin package[1] based on the procedure for mixed linear models described by Nakagawa and colleagues[2,3]. As for to the 4 time bins model, the use of a first-order polynomial (linear term) to model change in thought recall across spatial segments compared to a baseline (random intercept only) model did not improve model fit [$\chi^2(1) = 2.29$, $p = .13$]. However, the use of a second-order polynomial (quadratic term) improved model fit [$\chi^2(1) = 5.44$, $p = .02$], showing primacy and recency effects. Adding the effect of recall type [$\chi^2(1) = 0.12$, $p = .73$] and the interactions between recall type and the polynomial terms did not improve model fit [$\chi^2(1) = 0.05$, $p = .82$ for the interaction with the linear term; $\chi^2(1) = 0.003$, $p = .96$ for the interaction with the quadratic term], indicating that the primacy and recent effects did not differ between the free and cued recall phases (see **Figure S1**). The coefficients, standard errors, *t* and *p*-values for the fixed effects of the optimal (quadratic) model are presented in **Table S1**. The Marginal R^2 (i.e., the proportion of variance explained by the fixed effects[2,3]) for

the 4 time bins model was .026 and the corresponding value for the 3 spatial segments model was .016, indicating that the former explained more variance than the latter.

Table S1. Fixed effects of the optimal (quadratic) model for the growth curve analysis on the number of retrieved thoughts over the 3 spatial segments

	b	*SE*	*df*	*t-value*	*p-value*
Intercept	0.37	0.03	44	14.56	< .001
Linear term	-0.03	0.02	176	-1.54	.12
Quadratic term	0.05	0.02	176	2.35	.02

Note: participants and participants by recall type variability were included in the models as random effects.

Figure S1. Temporal distribution of recalled thoughts by spatial segments of the walk. Colored lines represent the fitted quadratic term. Error bars represent the standard error of the mean.

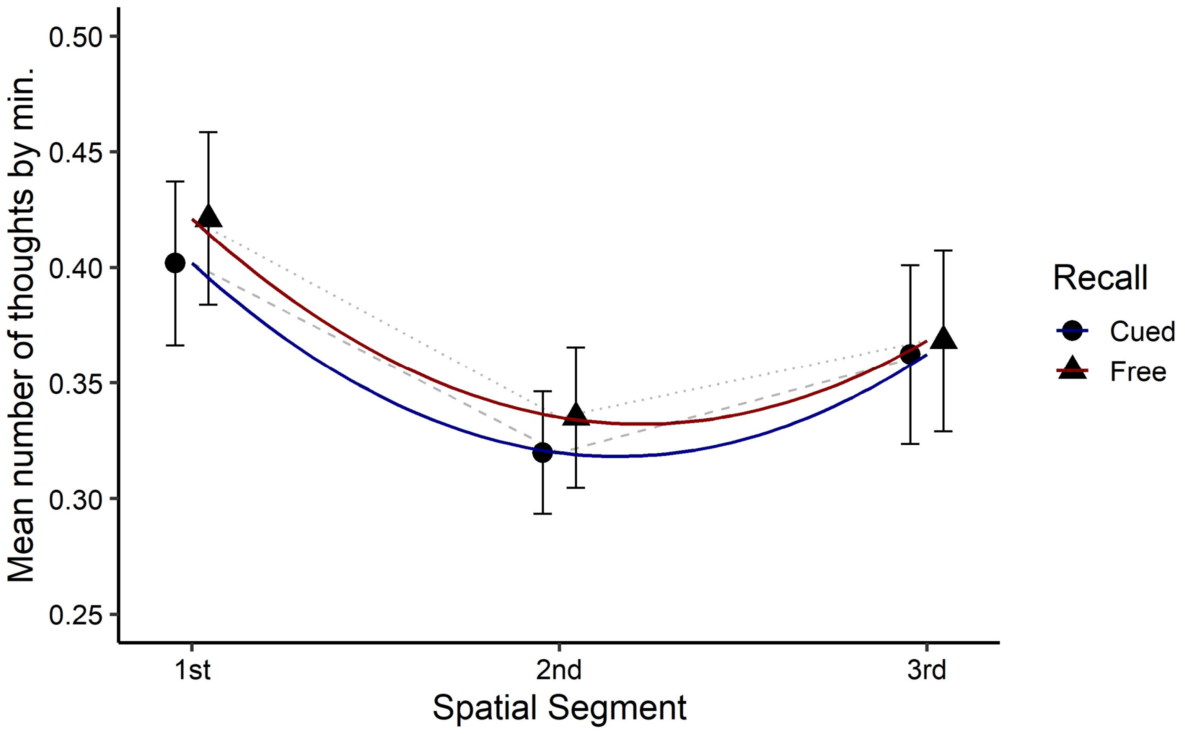

2. Number of retrieved thoughts in the periods preceding and following event boundaries.

As indicated in the main text, the number of retrieved thoughts increased with temporal proximity to event boundaries (i.e., the two actions). We further examined whether this effect was similar for the time bins preceding and following actions. To do so, the numbers of recalled thoughts were computed separately for the 2 minutes preceding and following each action, aggregated in four 30-second time bins (i.e., 30 s before/after the action; 30 to 60 s before/after the action; 60 to 90 s before/after the action; and 90 to 120 s before/after the action). Thoughts that occurred at the time of the actions (17 thoughts in the free recall task, and 8 thoughts in the cued recall task) were excluded from this analysis; furthermore, data from the cued and free recall task were pooled together because there were not enough thoughts in each time bin to analyze them separately for the two recall tasks. We then computed a growth curve analysis similar to the one presented in the main manuscript, except that we added a factor coding for the position of thoughts relative to the actions (i.e., before vs. after the actions) as a fixed effect. Results showed that adding the linear time term to the intercept only model improved model fit [$\chi^2(1) = 4.66$, $p = .03$], indicating that the linear decrease in the number of recalled thoughts with increased temporal distance from actions described in the main manuscript remained significant after excluding recalled thoughts that occurred right at the time of the action. Adding the quadratic term did not improve model fit [$\chi^2(1) = 0.03$, $p = .86$]. Adding the position of occurrence relative to the actions (before vs. after) did not improve the model fit [$\chi^2(1) = 0.09$, $p = .76$] and this term did not significantly interact with the linear [$\chi^2(1) = 2.02$, $p = .16$] or quadratic [$\chi^2(1) = 2.64$, $p = .10$] terms. Overall, these results indicate that the linear decrease in the number of recalled thoughts with increasing temporal

distance from the event boundaries did not differ between the 2 minutes preceding vs following actions **(See Figure S2 and Table S2).**

Table S2. Fixed effects of the optimal (linear) model for the growth curve analysis on the number of retrieved thoughts in the 2 minutes preceding and following the actions

	b	*SE*	*df*	*t-value*	*p-value*
Intercept	0.78	0.07	44	11.61	< .001
Linear term	0.21	0.09	308	2.17	.03

Note: Only participants were included as random effect in these models because the inclusion of participants by recall type variability in the random effects structure showed a singular model.

Figure S2. Distribution of recalled thoughts according to temporal distance from the actions (event boundaries). Colored lines represent the fitted linear term. Error bars represent the standard error of the mean. TRA = Time Relative to the Actions.

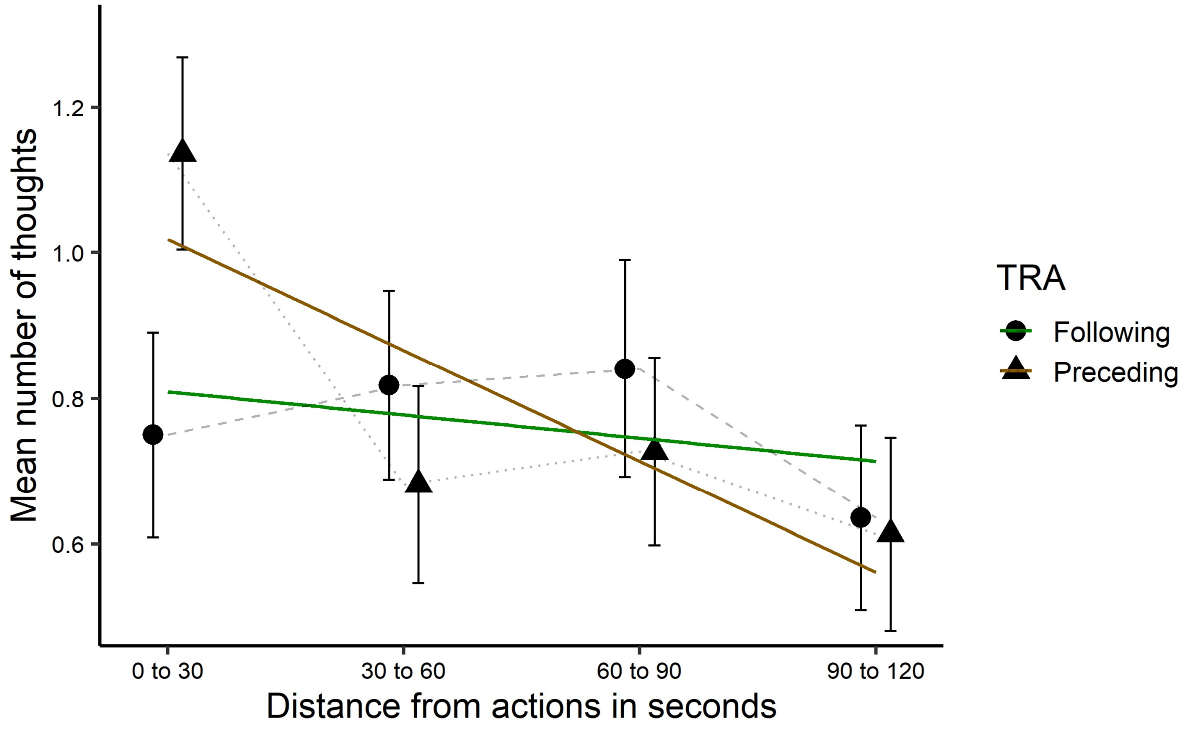

2. Number of future-oriented and planning thoughts retrieved over the 4 time bins

As future-oriented and planning thoughts were two categories of internal mentation of particular interest in the present study, we further examined whether their frequency varied with the 4 time bins of the walk, using growth curve analyses (note that given the low number of thoughts recalled in each of these categories we could not investigate whether their frequency increased with temporal proximity to the actions). For future-oriented thoughts, none of the terms improved model fit (all *p*s > .14) indicating that the number of retrieved future-oriented thoughts did not differ as a function of time bins (see **Figure S3**).

Figure S3. Distribution of future-oriented thoughts retrieved for each time bin of the walk. Colored lines represent the fitted quadratic term. Error bars represent the standard error of the mean.

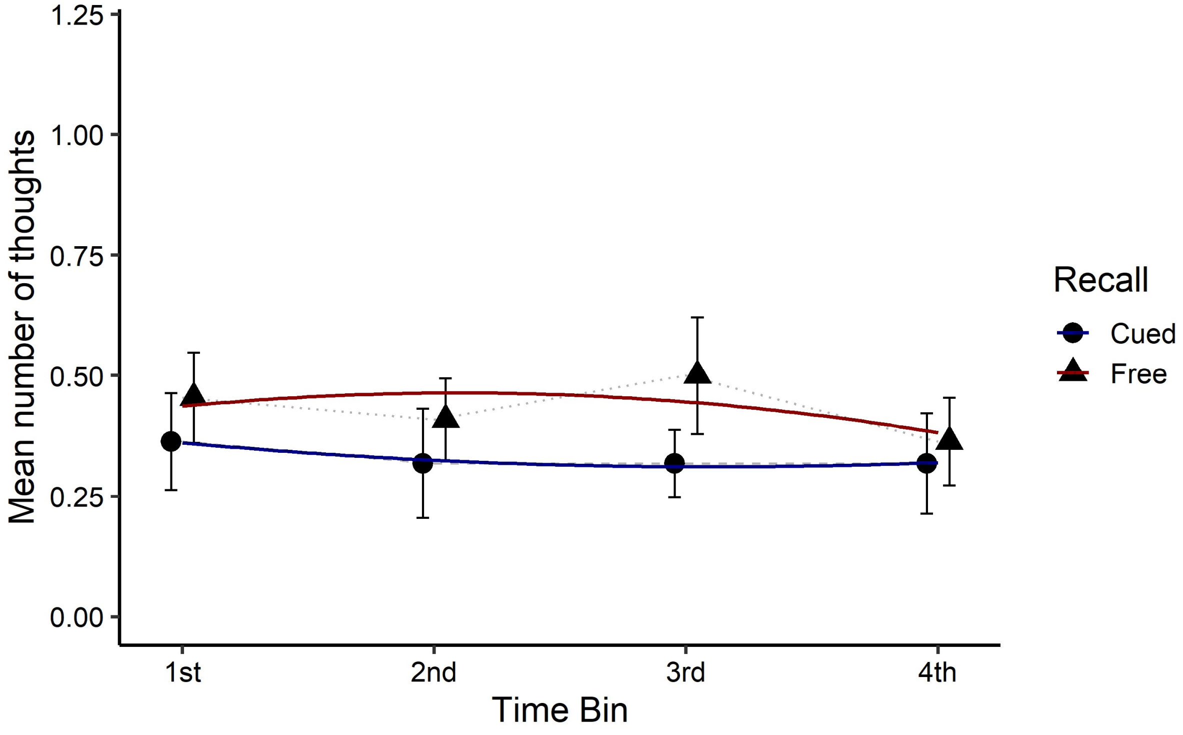

For planning-related thoughts, the linear term improved model fit [$\chi^2(1) = 6.77$, $p = .009$], indicating that more planning thoughts were retrieved from the beginning of the walk. The quadratic term also improved model fit [$\chi^2(1) = 6.14$, $p = .01$], indicating a steeper initial decrease in the number of recalled planning thoughts (see **Figure S4**). The recall type also improved model fit [$\chi^2(1) = 18.16$, $p < .001$], confirming that more thoughts were recalled in the free than cued recall task. None of the interactions between recall type and the time terms were significant [$\chi^2(1) = 0.01$, $p = .92$, for the linear terms, and $\chi^2(1) = 0.26$, $p = .61$, for the quadratic term]. The coefficients, standard errors, *t* and *p*-values for the fixed effects of the optimal model of each dimension are presented in **Table S3**.

Table S3. Fixed effects of the optimal model for the growth curve analysis on the number of planning thoughts retrieved thoughts over 4 time bins

	b	*SE*	*df*	*t-value*	*p-value*
Intercept	0.34	0.06	117.34	5.61	< .001
Linear term	-0.21	0.08	308.00	-2.72	.006
Quadratic term	0.20	0.08	308.00	2.57	.01
Recall type	0.34	0.08	308.00	4.33	< .001

Note: Only participants were included as random effect in these models because the inclusion of participants by recall type variability in the random effects structure showed a singular model fit.

Figure S4. Distribution of planning-related thoughts retrieved for each time bin of the walk. Colored lines represent the fitted quadratic term. Error bars represent the standard error of the mean.

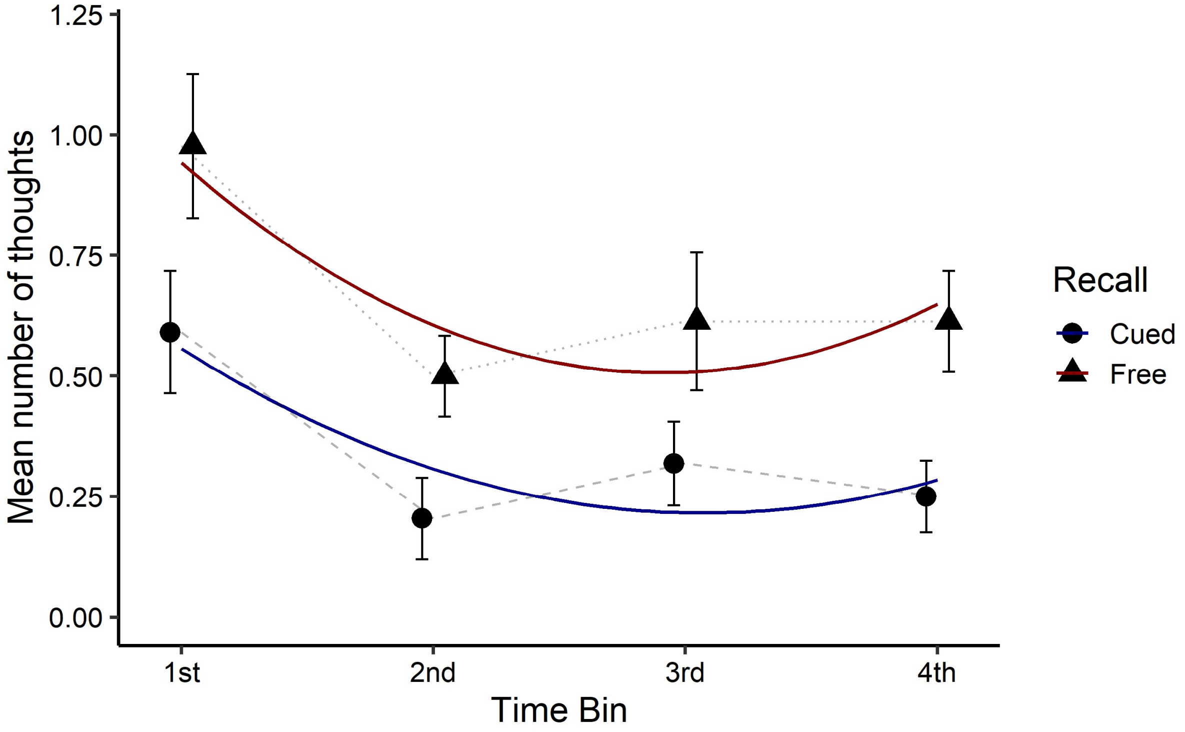

3. Phenomenological features of planning vs. non-planning thoughts

Table S4. Mean ratings for the characteristics of retrieved thoughts and two-tailed paired *t*-tests on differences between planning and non-planning thoughts.

Dimension	Planning *M* [95% CI]	Non-planning *M* [95% CI]	*t(41)*	*p*	*Cohen's d*
1. Deliberate	3.52 [3.07, 3.98]	2.56 [2.19, 3.93]	**4.61**	**<.001**	**0.71**
2. Walk-related	4.63 [4.11, 5.15]	3.62 [3.21, 4.03]	**3.12**	**.003**	**0.48**
3. Stimulus-dependence	4.43 [3.97, 4.89]	4.79 [4.45, 5.14]	-1.44	.16	-0.22
4. Visual format	4.25 [3.76, 4.75]	3.98 [3.56, 4.91]	1.16	.25	0.18
5. Inner speech format	4.56 [4.07, 5.05]	4.48 [4.09, 4.87]	0.45	.65	0.07
6. Affective valence	0.21 [-0.07, 0.50]	0.14 [-0.01, 0.30]	0.37	.71	0.06
7. Life frequency	3.30 [2.89, 3.72]	2.54 [2.25, 2.82]	**3.70**	**<.001**	**0.57**
8. Personal importance	2.81 [2.39, 3.23]	2.29 [1.98, 2.61]	1.88	.07	0.29
9. Specific/concrete	5.42 [5.03, 5.80]	4.86 [4.49, 5.22]	**3.36**	**.002**	**0.52**
10. Self-related	4.24 [3.77, 4.72]	3.52 [3.22, 3.83]	**3.61**	**<.001**	**0.56**
11. Other related	3.03 [2.50, 3.57]	3.86 [3.61, 4.11]	**-3.04**	**.004**	**0.47**
12. Unusual	2.70 [2.31, 3.10]	3.08 [2.75, 3.41]	**-2.10**	**.04**	**0.32**
13. Suppression	1.82 [1.46, 2.18]	1.65 [1.41, 1.88]	1.01	.32	0.16
14. Walk Freq. / Time in mind	3.29 [2.93, 3.64]	2.66 [2.43, 2.89]	**3.69**	**< .001**	**0.57**

Note: Each dimension ranged from 1 to 7, except for the affective valence dimension the range of which went from −3 to +3. Two participants did not rate any of their thoughts as fulfilling a planning function and were excluded from the analyses.

4. Modulation of thought features over the 4 time bins

We assessed whether the differences in thought characteristics between the two recall tasks varied depending on when the thoughts occurred during the walk. To do so, we performed a series of growth curve analyses with 4 time bins (similarly to the analyses reported in the main manuscript) with scores on each rating scale as dependent variable. For conciseness, only the significant effects are reported below (the data and fitted curves for all dimensions are shown on **Figure S5**).

For the **deliberate** dimension, the addition of the interaction between the linear term and recall type improved model fit [$\chi^2(1) = 6.99$, $p = .008$], indicating that in the free recall task the retrieved thoughts were judged to be more involuntary in later time bins whereas the cued recall task showed the opposite trend. For **stimulus-dependence**, the linear term [$\chi^2(1) = 5.88$, $p = .02$] and the interaction between the linear term and recall type [$\chi^2(1) = 4.57$, $p = .02$] improved model fit, indicating that in the free recall task retrieved thoughts tended to become less stimulus-dependent in later time bins whereas this was less the case for the cued recall task. For **inner speech format**, both the linear term [χ^2 (1) = 4.02, $p = .045$] and recall type [χ^2 (1) = 4.03, $p = .03$] improved model fit, indicating that retrieved thoughts from later time bins involved less inner speech and that the freely recalled thoughts involved more inner speech than the cued recalled thoughts. For the **life frequency** dimension, the quadratic term improved model fit [$\chi^2(1) = 6.09$, $p = .01$], although variations across time bins were quite small (see Figure S2). For **personal importance,** the effect of recall type improved model fit [$\chi^2(1) = 3.85$, $p = .049$], replicating the results of the *t*-test that freely recalled thoughts were rated as more personally important cued recalled thoughts. However, the interaction between the quadratic term and recall type [χ^2 (1) = 9.87, $p = .002$] also improved model fit, indicating that the difference in ratings between the two recall tasks was more marked

for the beginning than end of the walk. For the **unusualness** of content and **suppression** attempt dimensions, the effect of recall type improved model fit [$\chi^2(1) = 10.93$, $p = .001$, and $\chi^2(1) = 7.57$, $p = .01$, respectively], indicating higher ratings on these two dimensions for the free than cued recall tasks (in line with the analyses reported in the main manuscript). Finally, for the **time in mind and frequency of occurrence** during the walk, both the linear [χ^2 (1) = 13.06, $p < .001$] and quadratic [χ^2 (1) = 13.57, $p < .001$] terms improved model fit, as well as the recall type [χ^2 (1) = 53.50, $p < .001$] and the interaction between recall type and the quadratic terms [χ^2 (1) = 11.47, $p = .001$]. These results indicate that, overall, the retrieved thoughts were rated as more frequently occurring in mind during the walk in the free than cued task, and this difference decreased with increasing time spent in the walk. More specifically, scores decreased linearly in the free recall task whereas they showed a quadratic increase in the last bin after an initial decrease for the cued recall task. The coefficients, standard errors, *t* and *p*-values for the fixed effects of the optimal model for each of these analyses are presented in **Table S5** with **Figure S5** illustrating the data and fitted quadratic curves for each dimension.

In conclusion, these analyses showed that some dimensions of recalled thoughts differed depending on their time of occurrence during the walk, but most effects were of small amplitude (see **Figure S5**). The results nonetheless showed that differences between the two recall tasks in the personal importance, time in mind/frequency of occurrence during the walk, and to a lesser extent the deliberate dimension of thoughts were larger at the beginning than end of the walk.

Table S5. Fixed effects for the growth curve analyses investigating the modulation of thought ratings over the 4 time bins

Dimensions	Fixed effects	*b*	*SE*	*df*	*t-value*	*p-value*
**Deliberate**	Intercept	2.71	0.18	58.22	14.95	< .001
	Linear term	0.41	0.19	765.61	2.17	.03
	Quadratic term	0.24	0.13	763.55	1.81	.07
	Recall type	0.16	0.13	773.92	1.20	.23
	Linear term by recall type	-0.70	0.26	764.41	-2.65	.008
**Stimulus-dep.**	Intercept	4.74	0.16	62.78	28.96	< .001
	Linear term	-0.04	0.18	769.29	-0.25	.80
	Quadratic term	-0.10	0.12	767.23	-0.82	.41
	Recall type	-0.13	0.13	777.92	-1.07	.29
	Linear term by recall type	-0.53	0.25	768.07	-2.14	.03
**Inner speech**	Intercept	4.30	0.19	55.44	22.55	< .001
**Format**	Linear term	-0.24	0.12	764.84	-1.93	.053
	Quadratic term	0.08	0.12	762.03	0.66	.51
	Recall type	0.26	0.12	769.47	2.14	.03
**Life Frequency**	Intercept	2.76	0.13	45.87	21.21	< .001
	Linear term	-0.07	0.13	775.71	-0.50	.61
	Quadratic term	0.32	0.13	769.60	2.47	.01
**Personal**	Intercept	2.28	0.14	66.59	16.56	< .001
**Importance**	Linear term	0.03	0.18	772.75	0.15	.88
	Quadratic term	-0.30	0.17	770.73	-1.74	.08
	Recall type	0.24	0.12	785.31	1.93	.054
	Linear term by recall type	-0.14	0.24	771.44	-0.57	.57
	Quadratic term by recall type	0.76	0.24	768.98	3.15	.002
**Unusual**	Intercept	2.68	0.16	65.90	16.24	< .001
	Linear term	< 0.001	0.13	773.80	0.01	.99
	Quadratic term	0.05	0.13	769.20	0.39	.70
	Recall type	0.44	0.13	780.40	3.32	< .001
**Suppression**	Intercept	1.54	0.11	62.00	13.61	< .001
	Linear term	0.06	0.09	770.96	0.73	.47
	Quadratic term	- 0.002	0.08	766.99	-0.03	.98
	Recall type	0.24	0.09	776.82	2.76	.01

**Walk freq./Time in mind**	Intercept	2.35	0.12	62.80	19.62	< .001
	Linear term	-0.27	0.14	770.03	-1.86	.06
	Quadratic term	0.04	0.14	768.27	0.26	.79
	Recall type	0.75	0.10	781.50	7.41	< .001
	Linear term by recall type	-0.15	0.20	768.98	-0.76	.45
	Quadratic term by recall type	0.67	0.20	766.68	3.40	< .001

Note: These analyses were performed on the 800 thoughts associated with a single image (nested within the 44 participants). Only participants were included as random effect in these models because the inclusion of participants by recall type variability in the random effects structure showed a singular model fit.

Figure S5. Temporal distribution for each dimension of mean thought rating for each time bin of the walk. Colored lines represent the quadratic terms. Error bars represent the standard error of the mean.

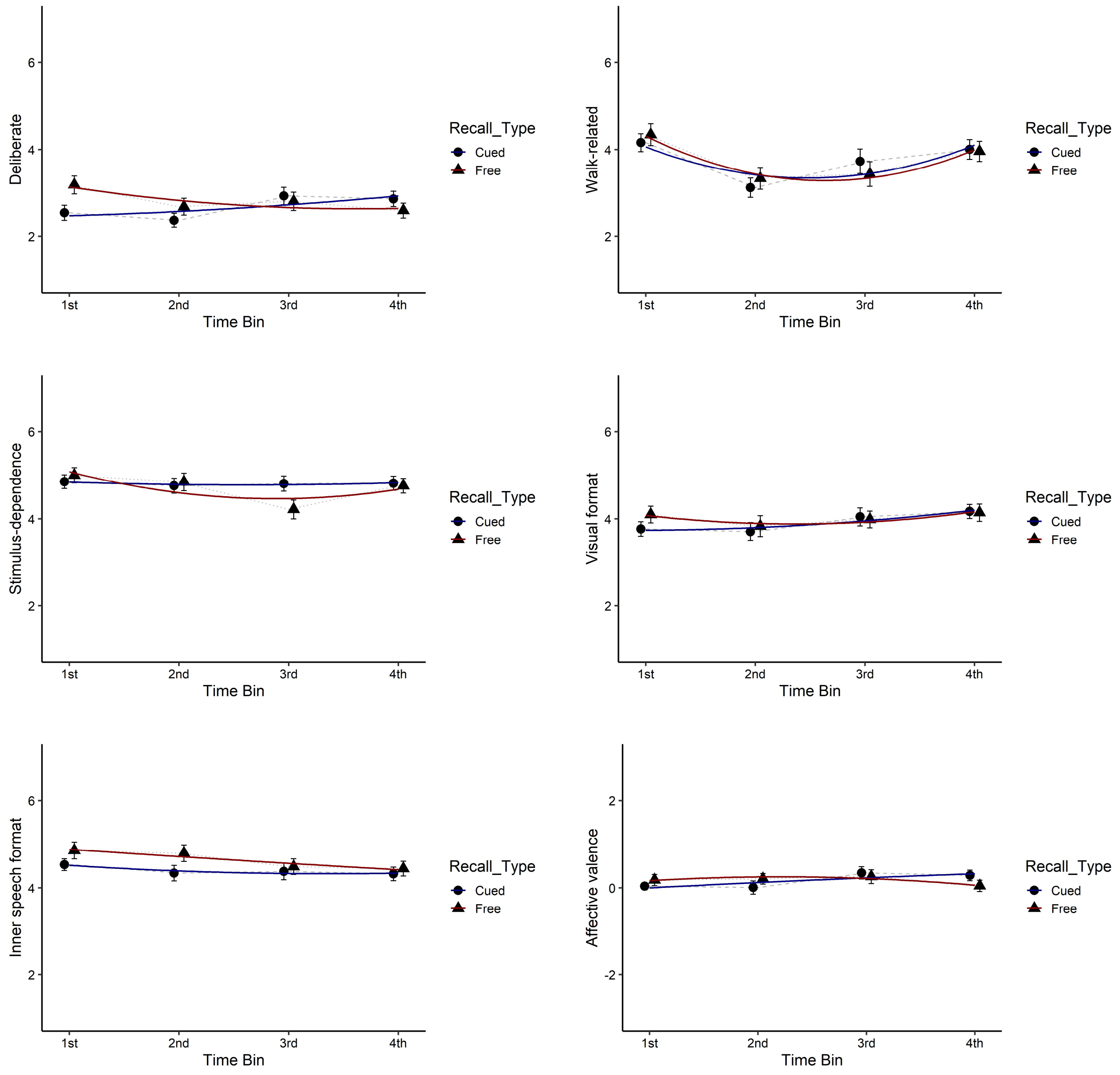

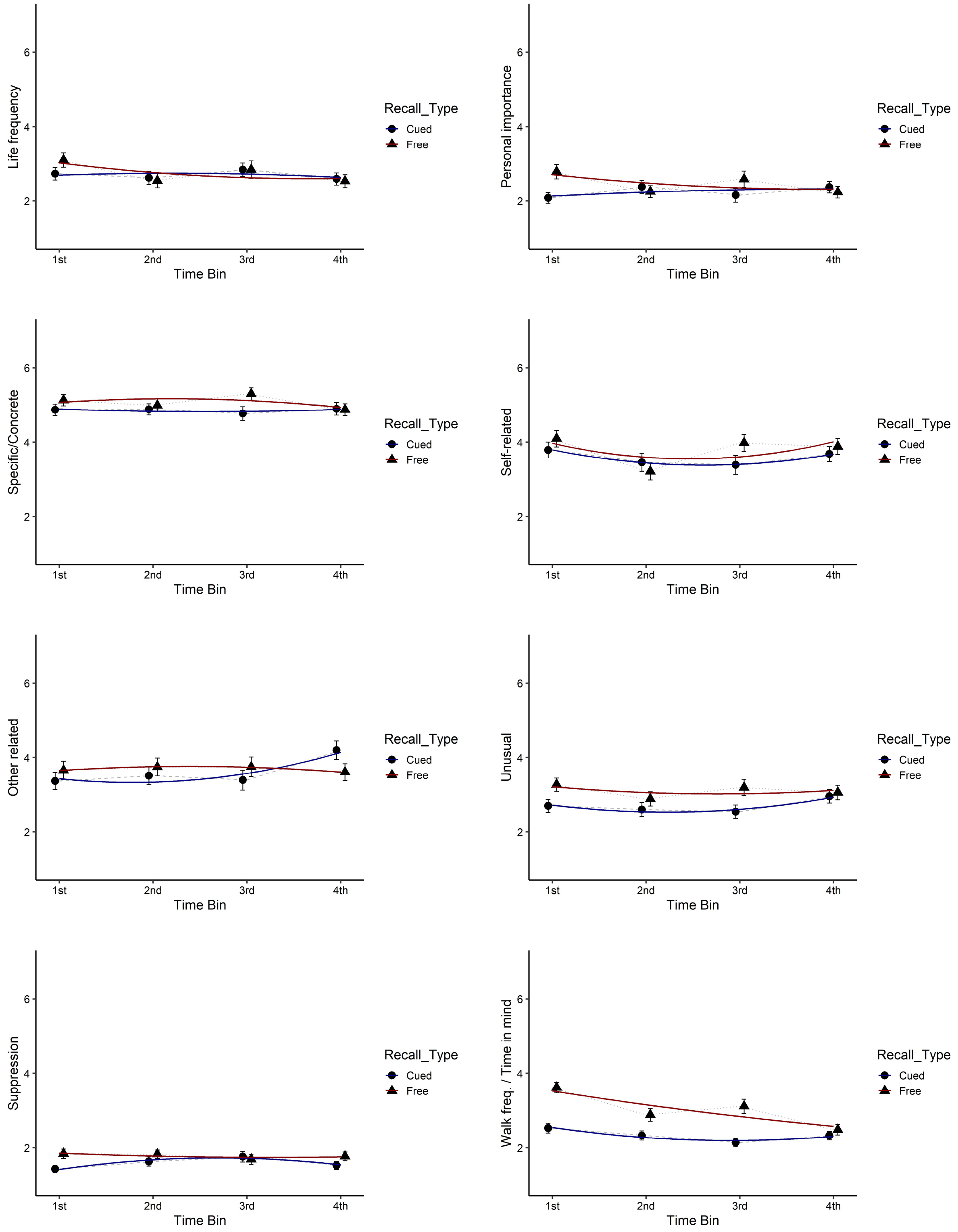
Life frequency
Personal importance
Specific/Concrete
Self-related
Other related
Unusual
Suppression
Walk freq. / Time in mind
Time Bin
1st
2nd
3rd
4th
Recall_Type
Cued
Free

5. Modulation of thought ratings with temporal distance from the actions

We assessed whether differences in thought characteristics varied between the two recall tasks depending on their temporal proximity from the actions defining event boundaries. We divided the four minutes surrounding the action in four 1-minute time bins and computed growth curve analyses with scores on the Likert scales as dependent variable. These analyses were performed on the 298 thoughts associated with a single picture (nested within 43 participants) that were reported as having occurred in these 4 minutes of interest. For conciseness, only the significant improvement of model fit for each dimension are reported below.

For the **walk-relatedness** dimension, only the quadratic term improved model fit [$\chi^2(1) = 4.02$, $p = .04$] indicating that walk-relatedness decreased from the first time bin but then increased in the later time bins. For **inner speech**, ratings were higher for the free than cued recall task, as indicated by an improvement of model fit with recall type [$\chi^2(1) = 10.07$, $p = .002$]. For the **life frequency** dimension, the linear term improve model fit [$\chi^2(1) = 11.71$, $p < .001$], indicating that scores decreased with temporal proximity from the actions and the interaction between recall type and the linear term also improved model fit [$\chi^2(1) = 3.96$, $p = .047$], indicating that this decrease was steeper for the cued than free recall task. For the **unusual** dimension, only the recall type improved the model fit [$\chi^2(1) = 6.64$, $p = .01$], indicating that retrieved thoughts had more unusual content in the free than cued recall task. For **suppression attempts**, the interaction between the quadratic term and recall type improved model fit [$\chi^2(1) = 4.73$, $p = .03$], indicating that ratings showed a positive (u-shaped) curve in the free and negative curve in the cued recall task. Finally, for the **time in mind and frequency of occurrence** during the walk, recall type improved model fit [$\chi^2(1) = 13.70$, $p < .001$] indicating that retrieved thoughts were more recurrent in the free than cued recall task. The coefficients, standard errors, *t* and *p*-values for the fixed effects of the optimal model of each

dimension are presented in **Table S6** with **Figure S6** illustrating the data and fitted quadratic curves.

In conclusion, these analyses suggest that some dimensions of recalled thoughts varied with their temporal proximity to event boundaries (i.e., actions) but most effects were of small amplitude (see **Figure S6**). The retrieved thoughts were more related to the walk and occurred less frequently in daily life (particularly for the cued recall task) with increasing temporal proximity from the boundaries.

Table S6. Fixed effects for the growth curve analyses investigating the modulation of thought ratings on the Likert scales with temporal proximity to the actions.

Dimensions	Fixed effects	*b*	*SE*	*df*	*t-value*	*p-value*
**Walk-related**	Intercept	3.91	0.20	39.09	19.30	< .001
	Linear term	-0.12	0.28	289.51	-0.43	.67
	Quadratic term	0.59	0.29	291.43	2.02	.04
**Inner speech format**	Intercept	3.96	0.25	64.43	15.84	< .001
	Linear term	-0.24	0.20	273.04	-1.16	0.25
	Quadratic term	0.21	0.21	272.41	1.02	0.31
	Recall type	0.26	0.12	769.47	2.14	.03
**Life Frequency**	Intercept	2.66	0.20	84.98	13.34	< .001
	Linear term	1.10	0.30	275.08	3.65	< .001
	Quadratic term	-0.10	0.21	286.55	-0.47	.64
	Recall type	-0.21	0.21	287.36	-1.02	.31
	Linear term by recall type	-0.79	0.40	279.54	-2.00	.046
**Unusual**	Intercept	2.49	0.22	88.88	11.28	< .001
	Linear term	-0.07	0.23	287.20	-0.33	0.74
	Quadratic term	-0.31	0.23	288.08	-1.35	0.18
	Recall type	0.58	0.23	287.26	2.59	0.01
**Suppression**	Intercept	1.54	0.14	79.68	10.92	< .001
	Linear term	0.11	0.19	271.44	0.57	.57
	Quadratic term	-0.20	0.19	270.95	-1.02	.31
	Recall type	0.11	0.13	281.98	0.81	.42
	Linear term by recall type	0.12	0.26	277.14	0.45	.65
	Quadratic term by recall type	0.56	0.26	271.97	2.19	.03
**Walk freq./Time in mind**	Intercept	2.12	0.15	94.43	13.89	< .001
	Linear term	0.24	0.17	291.64	1.44	.15
	Quadratic term	-0.09	0.17	293.25	-0.52	.61
	Recall type	0.63	0.17	292.09	3.75	< .001

Note: These analyses were performed on the 298 thoughts associated with a single picture (nested within 43 participants) that were reported as having occurred in the 4 minutes of interest. Only participants were included as random effect in these models because the inclusion of participants by recall type variability in the random effects structure showed a singular model fit.

Figure S6. Distribution of mean ratings for each dimension according to temporal distance from the actions. Colored lines represent the quadratic terms. Error bars represent the standard error of the mean.

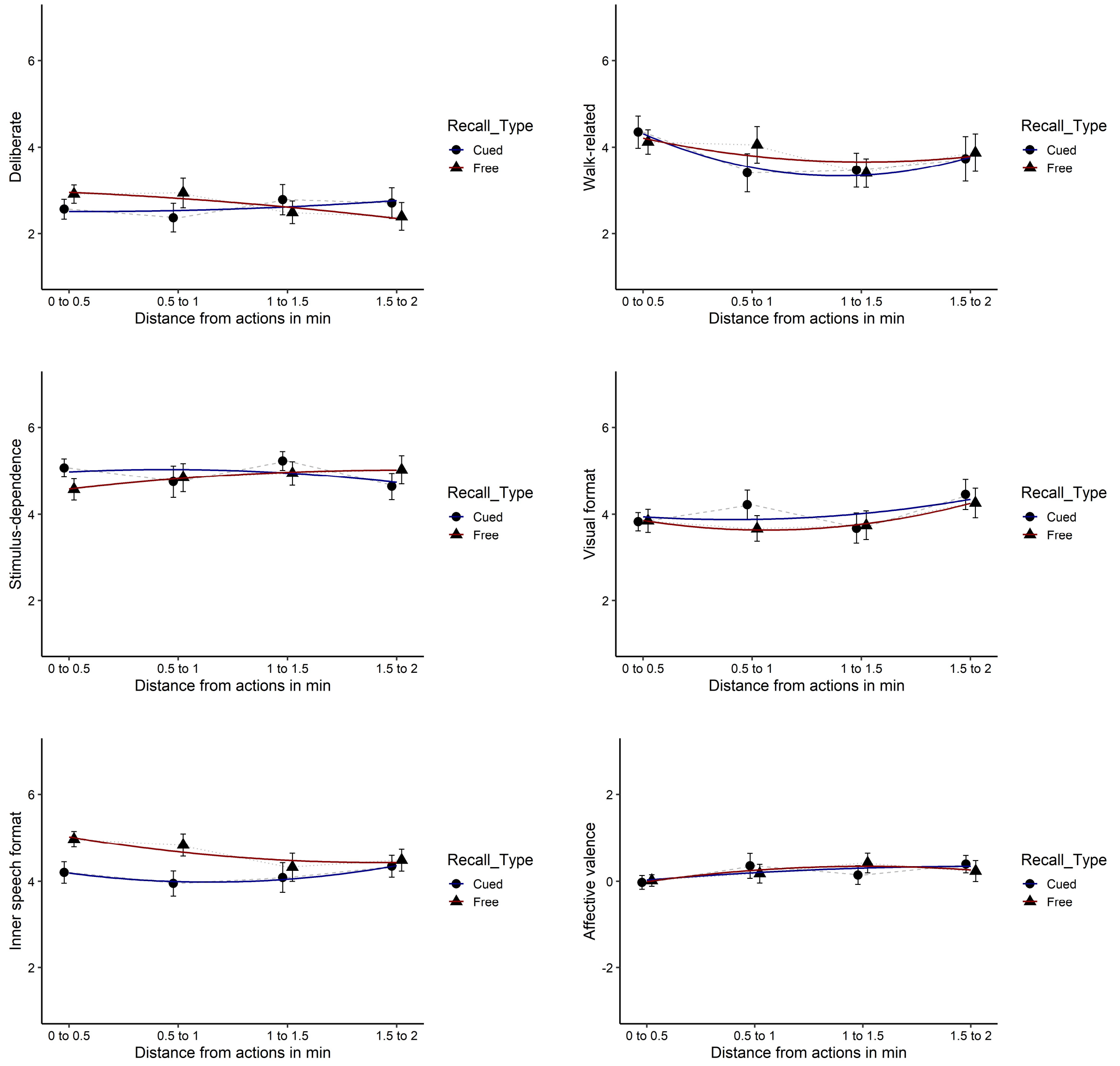

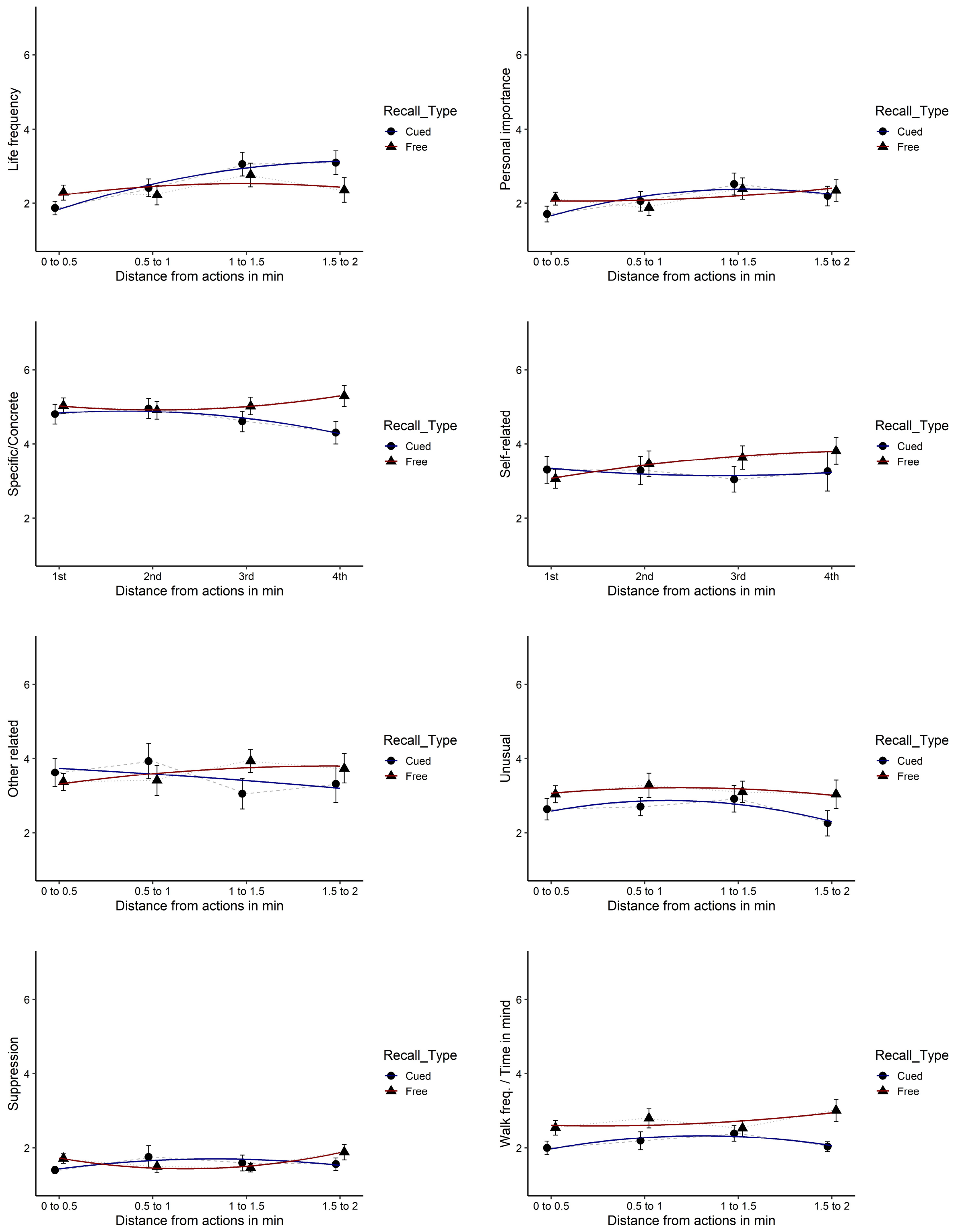
Life frequency
Personal importance
Specific/Concrete
Self-related
Other related
Unusual
Suppression
Walk freq. / Time in mind
6
4
2
0 to 0.5
0.5 to 1
1 to 1.5
1.5 to 2
1st
2nd
3rd
4th
Distance from actions in min
Recall_Type
Cued
Free

6. Ease of retrieval of thoughts as a function of the 4 time bins

We examined whether ratings on the ease of retrieval of thoughts during the free recall task varied with their time of occurrence during the walk. To do so, we performed growth curve analyses with 4 time bins (similarly to the analyses reported in the main manuscript) with scores on the ease of retrieval scale as dependent variable and first-order and second-order polynomials as fixed effects. Ratings were missing for three participants and we therefore computed the models on the 379 thoughts (with a single time of occurrence) freely recalled by the remaining 41 participants. Results revealed that the linear term improved model fit [$\chi^2(1) = 16.55$, $p < .001$], as did the quadratic term [$\chi^2(1) = 8.41$, $p < .001$], showing that ease of retrieval decreased with time in the walk and that this decrease was steeper in the first time bins (see **Figure S7**.) The coefficients, standard errors, *t* and *p*-values for the fixed effects of the optimal model are presented in **Table S7**.

Table S7. Fixed effects of the optimal model for the growth curve analysis investigating the modulation of ease of thought retrieval over the 4 time bins

	b	*SE*	*df*	*t-value*	*p-value*
Intercept	5.29	0.16	38.44	32.19	< .001
Linear term	-0.61	0.16	352.86	-3.86	< .001
Quadratic term	0.45	0.16	348.17	2.92	.004

Note: ratings on this scale were missing for three participants and we therefore computed the models on the 379 freely recalled thoughts that could be associated with a single picture by the remaining 41 participants. Participants were included as random effect in these models.

Figure S7. Temporal distribution for the ease of retrieval of thoughts for each time bin of the walk. The colored line represents the fitted quadratic term. Error bars represent the standard error of the mean.

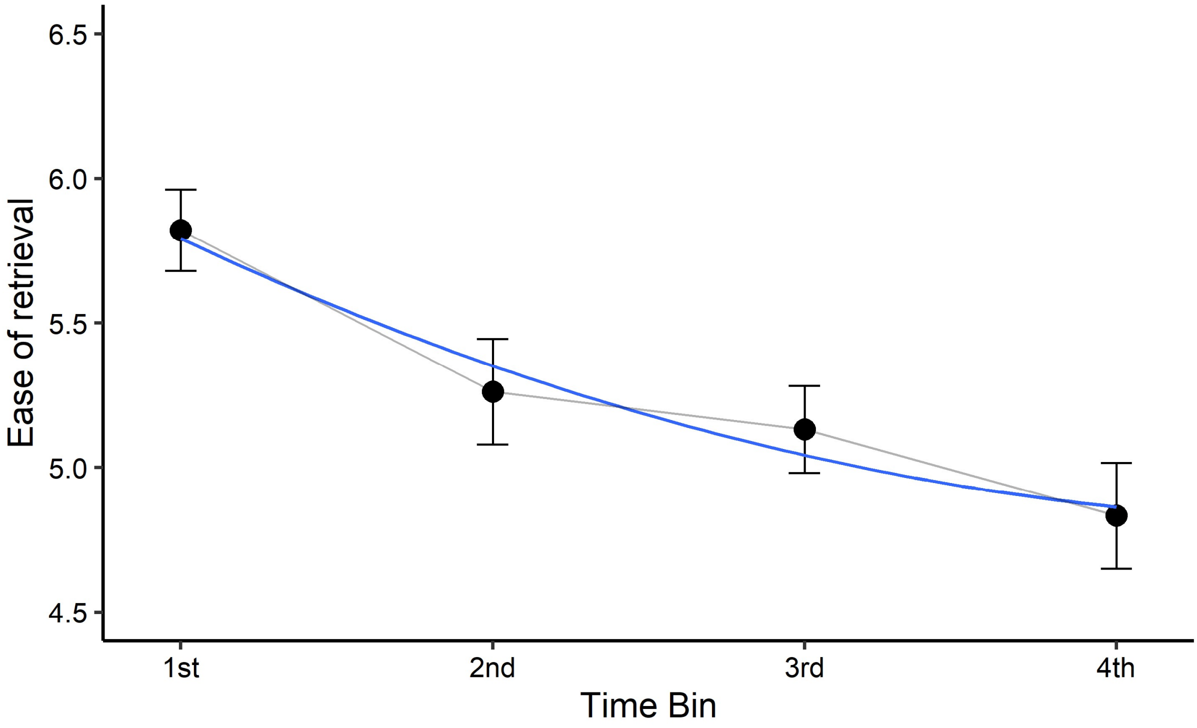

Table S8. Maximum likelihood estimated sigma within-subject correlation matrix

	1.	2.	3.	4.	5.	6.	7.	8.	9.	10.	11.	12.	13.	14.	15.
1. Deliberate	1.00														
2. Walk-related	.272	1.00													
3. Stimulus-dep.	-.202	.045	1.00												
4. Visual	-.006	-.066	-.022	1.00											
5. Inner speech	.062	.131	.066	**-.389**	1.00										
6. Affective val.	-.019	-.200	-.138	.207	-.152	1.00									
7. Freq. walk	.125	.144	.073	-.040	0.150	-.029	1.00								
8. Freq. life	-.057	-.175	.106	-.014	.027	.091	.231	1.00							
9. Personal imp.	.001	-.172	-.008	.066	.043	.126	.201	**.442**	1.00						
10. Specific	.096	.063	-.060	.006	.052	.012	.034	-.085	.163	1.00					
11. Self-related	-.020	.065	.082	-.039	.052	-.023	.189	.280	.261	.023	1.00				
12. Other related	-.085	-.275	-.044	-.010	-.016	.082	-.083	-.128	.140	.071	-.015	1.00			
13. Unusual	-.025	.029	-.039	.055	.071	.038	.106	-.207	.095	.247	.040	0.289	1.00		
14. Time in mind	.073	-.038	.016	-.019	.152	-.012	**.613**	.165	.239	.108	.182	.034	.238	1.00	
15. Suppression	-.002	-.024	.075	-.012	.110	-.197	.208	.030	.088	.076	.131	.009	.123	.262	1.00

Note: correlations above .30 and below -.30 are indicated in bold.
